# OPA1 promotes pH flashes that spread between contiguous mitochondria without matrix protein exchange

**DOI:** 10.1038/emboj.2013.124

**Published:** 2013-05-28

**Authors:** Jaime Santo-Domingo, Marta Giacomello, Damon Poburko, Luca Scorrano, Nicolas Demaurex

**Affiliations:** 1 Department of Cell Physiology and Metabolism, University of Geneva, Geneva, Switzerland; 2 Department of Biomedical Physiology and Kinesiology, Simon Fraser University, Burnaby, British Columbia, Canada

**Keywords:** bioenergetics, cell signalling, metabolism

## Abstract

The chemical nature and functional significance of mitochondrial flashes associated with fluctuations in mitochondrial membrane potential is unclear. Using a ratiometric pH probe insensitive to superoxide, we show that flashes reflect matrix alkalinization transients of ∼0.4 pH units that persist in cells permeabilized in ion-free solutions and can be evoked by imposed mitochondrial depolarization. Ablation of the pro-fusion protein Optic atrophy 1 specifically abrogated pH flashes and reduced the propagation of matrix photoactivated GFP (paGFP). Ablation or invalidation of the pro-fission Dynamin-related protein 1 greatly enhanced flash propagation between contiguous mitochondria but marginally increased paGFP matrix diffusion, indicating that flashes propagate without matrix content exchange. The pH flashes were associated with synchronous depolarization and hyperpolarization events that promoted the membrane potential equilibration of juxtaposed mitochondria. We propose that flashes are energy conservation events triggered by the opening of a fusion pore between two contiguous mitochondria of different membrane potentials, propagating without matrix fusion to equilibrate the energetic state of connected mitochondria.

## Introduction

Mitochondria are double-membrane organelles that play a central role in cellular energy conversion, lipid metabolism, calcium signalling, and apoptosis. The generation of ATP by oxidative phosphorylation involves the generation of a proton-motive force (Δ*p*) across the inner mitochondrial membrane (IMM) as protons are pumped by respiratory chain complexes and subsequently used to drive the activity of the ATP synthase. Δ*p* comprises an electrical component, the mitochondrial membrane potential (ΔΨ_m_ ∼180 mV, negative inside), and a chemical component, the transmembrane pH gradient (ΔpH_m_ ∼0.8, alkaline inside), whose generation is facilitated by the low H^+^-buffering capacity of the alkaline mitochondrial matrix (Poburko et al, 2011). While some electrogenic transporters are driven exclusively by ΔΨ_m_, the transport of many ions, substrates, and metabolites depends on ΔpH_m_ (Bernardi, 1999).

Improvements in live cell fluorescence imaging have revealed that ΔΨ_m_ fluctuates rapidly within individual mitochondria and that these electrical events can propagate along interconnected mitochondria (Duchen et al, 1998; Huser et al, 1998; Huser and Blatter, 1999; De Giorgi et al, 2000). A plethora of mechanisms were proposed to trigger the ΔΨ_m_ fluctuations: local Ca^2+^ elevations (Duchen et al, 1998), opening of the mitochondrial permeability transition pore (mPTP) (Huser and Blatter, 1999; De Giorgi et al, 2000; Zorov et al, 2000; Jacobson and Duchen, 2002), coupling of ΔΨ_m_ to the ATP synthase (Thiffault and Bennett, 2005), switching between active and inactive states of oxidative phosphorylation (Buckman and Reynolds, 2001), or opening of a proton-selective channel by matrix alkalinization (Hattori et al, 2005). Spontaneous ΔΨ_m_ fluctuations are also observed in permeabilized cells (Uechi et al, 2006) and in isolated mitochondria, where they are modulated by adenine nucleotides acting from the matrix side (Vergun et al, 2003; Vergun and Reynolds, 2004). In astrocytes, spontaneous ΔΨ_m_ decreases are associated with transient elevations in matrix [Na^+^] (Azarias et al, 2008), whereas in cardiac myocytes synchronized ΔΨ_m_, reactive oxygen species (ROS), and NADP fluctuations were reported and attributed to the opening of a mitochondrial anion channel permeable to superoxide (Aon et al, 2003). In skeletal muscle cells and intact beating hearts, superoxide flashes coinciding with ΔΨ_m_ decreases were recorded with a circularly permutated yellow fluorescent protein (cpYFP) and proposed to be generated by stochastic openings of the mPTP that, by dissipating ΔΨ_m_, divert electrons from the respiratory chain to generate bursts of matrix superoxide (Wang et al, 2008). Subsequent studies using cpYFP-based probes indicated that flash frequency is linked to mitochondrial respiration (Pouvreau, 2010; Wei et al, 2011) and increases during oxidative stress-induced apoptosis (Ma et al, 2011), reviewed in Fang et al (2011). The superoxide nature of the flashes is disputed, however (Muller, 2009), and because cpYFP is also pH sensitive (Nagai et al, 2001) several groups have instead proposed that the flashes are transient mitochondrial matrix pH (pH_mito_) elevations (Azarias and Chatton, 2011; Schwarzlander et al, 2011, 2012a), reviewed in Santo-Domingo and Demaurex (2012) and Schwarzlander et al (2012b).

Energy conservation across the IMM depends on its impermeability to protons; however, the maintenance of this permeability barrier is challenged in intact cells by the understanding that mitochondria are not isolated organelles and that they undergo cycles of fission and most importantly fusion (Twig et al, 2008). Mitochondrial fission depends on the cytoplasmic dynamin-related protein 1 (DRP1) (Smirnova et al, 2001), that is recruited on the organelle by several potential receptors like FIS1, MFF, and MID49/51 (Palmer et al, 2011). Fusion depends on the outer mitochondrial membrane (OMM) proteins Mitofusin (MFN) 1 and 2 and on the IMM Optic atrophy 1 (OPA1) (Campello and Scorrano, 2010). Mitochondrial fusion is a complex process from the membrane biology and the bioenergetic point of view: fusion of two organelles involves the generation of a fusion intermediate of four membranes; if the process of mitochondrial fusion is analogous to other organellar fusions, when the IMM fuses a fusion pore shall be generated that would link two matrixes. Such a fusion pore might connect two mitochondria of different respiratory states, with unpredictable effects on their membrane potential. Defective fusion pore assembly could even connect the matrix with the intermembrane space. *In vitro* experiments indicate that OPA1 induces lipid tubulation (Ban et al, 2010), and rupture of the growing IMM tubules could link the matrix to the IMS. If pore formation involves the juxtaposition of two hemichannels as for gap junctions, opening of the hemichannels on the growing IMM tubule would also connect the matrix to the IMS, equilibrating two chemically different environments with profound consequences on the bioenergetics of the organelle.

Here we set out to address, by combining genetics and physiology, how mitochondrial fusion impacts on bioenergetics. Our ratiometric probe SypHer, highly pH-sensitive but insensitive to superoxide *in vitro*, recorded changes in matrix pH in single mitochondria. Spontaneous alkalinization transients coincided with decreases in ΔΨ_m_. We could unravel that these flashes represented compensatory pH_mito_ elevations maintaining the proton-motive force during spontaneous decreases in ΔΨ_m_. In cells lacking OPA1, the flashes were completely absent, whereas their propagation was greatly increased when mitochondria were more interconnected. A significant fraction of adjacent mitochondria exhibited opposite changes in ΔΨ_m_ during pH_mito_ flashes that resulted in membrane potential equilibration. We propose that the pH_mito_ flashes are energy conservation events requiring the fusion protein OPA1 and therefore triggered by the opening of fusion pores between adjacent mitochondria. The flashes propagate without matrix mixing between adjacent mitochondria, a new mode of coupling that might allow interconnected mitochondria to rapidly equilibrate their energetic state.

## Results

### Spontaneous alkalinization transients in single mitochondria

**Figure 1:**
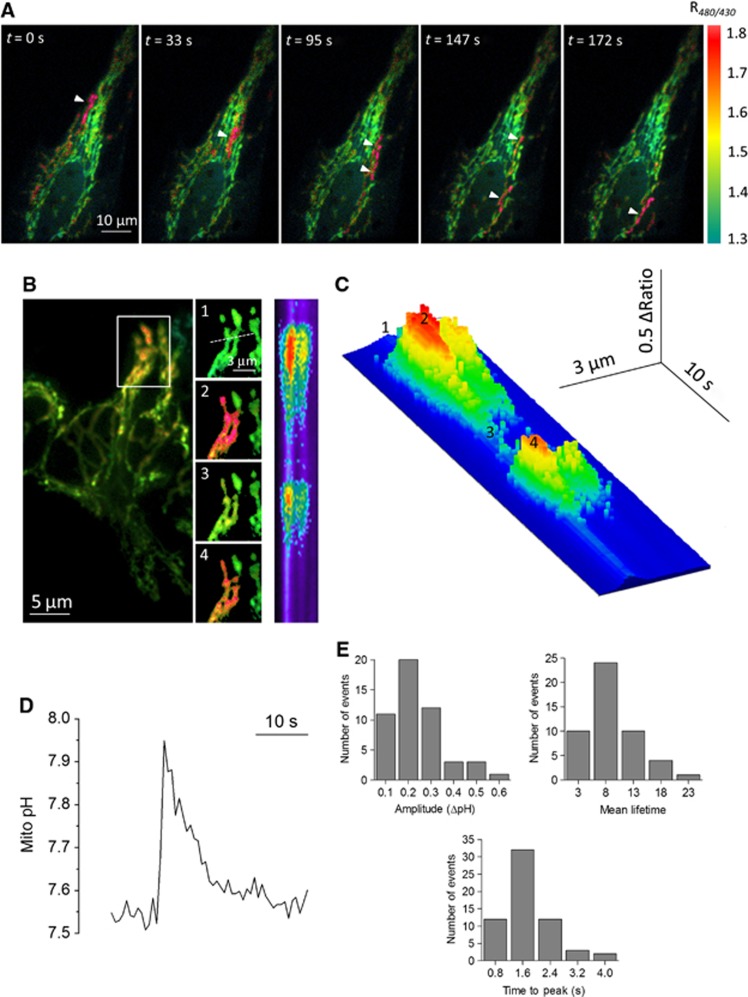
Transients pH_mito_ elevations in single mitochondria. (**A**) Time sequence of F480/F430 ratio images of HeLa cells expressing mito-SypHer showing spontaneous alkalinization transients (arrows) in single mitochondria in different cellular regions. Warm colours denote high ratio values. See also Supplementary Movie S1. (**B**) Repetitive pH transients in a mitochondrial cluster. Numbered insets show consecutive images and the right-hand panel shows a 30 s scan along the line drawn in inset #1, the time axis running vertically from top to bottom. (**C**) 3D reconstruction of the line scan image. Numbers correspond to inset panels in **B**. (**D**) Calibrated pH_mito_ transient. (**E**) Histograms showing the distribution of the amplitude, time to peak, and half-life time of 61 independent pH_mito_ elevations.

We recently generated a new pH-sensitive probe targeted to the matrix of mitochondria, mito-SypHer, and reported dynamic changes in the mitochondrial pH gradient in HeLa cells (Poburko et al, 2011). During these recordings, we frequently observed spontaneous and asynchronous increases in mito-SypHer ratio fluorescence in discrete regions of the mitochondrial network (Figure 1 and Supplementary Movie S1). The elevations occurred either in different regions of the cell (Figure 1A) or repeatedly at the same location (Figure 1B), but always remained restricted to a specific region of the mitochondrial network (Figure 1B, inset). The elevations had an abrupt onset (time to peak: 1.63±0.08 s) followed by a slower recovery towards basal levels, and a mean life time of 8.6±0.6 s (Figure 1C–E). On average, 0.54±0.04 elevations were detected per minute per cell. *In situ* pH calibration of the probe by titration with buffers of different pH in the presence of the K^+^/H^+^ ionophore nigericin (Supplementary Figure S1) revealed that the matrix pH increased by 0.38±0.04 pH units during a typical event, from 7.75±0.21 to 8.14±0.46 (Figure 1D and E). Transient alkalinization events of sizable magnitude thus occur in intact mitochondria.

**Figure 2:**
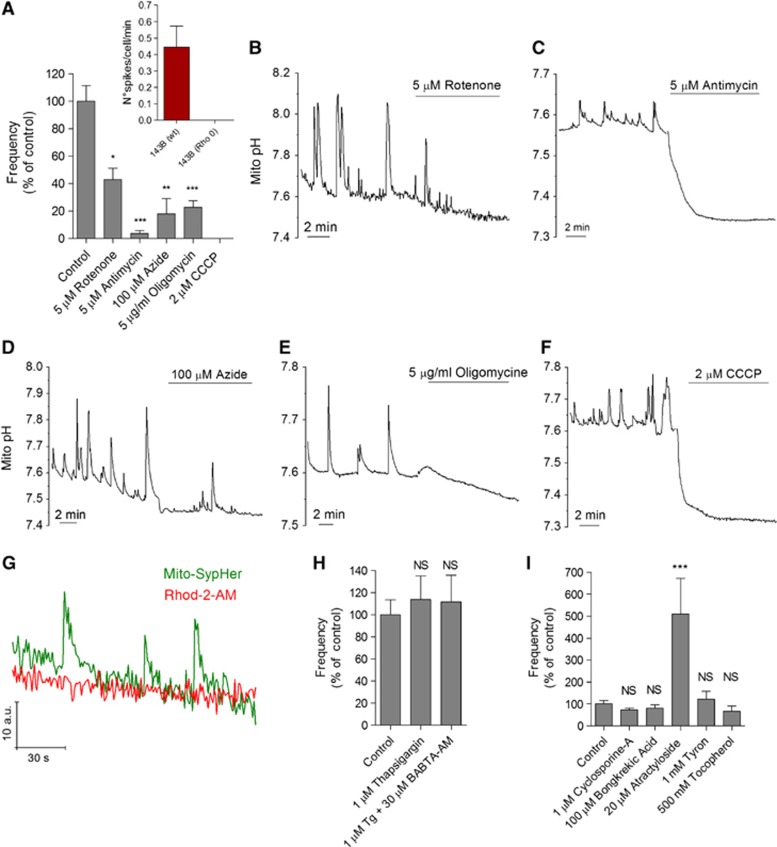
Pharmacology of pH_mito_ flashes. (**A**) Effect of respiratory chain inhibitors on the frequency of pH_mito_ flashes in 15 min recordings of 82 (control), 10 (rotenone), 28 (antimycin), 13 (azide), 48 (oligomycin), and 11 cells (CCCP). Inset: pH_mito_ elevations in control (*n*=14) and mitochondria-deficient *rho 0* osteosarcoma 143b cells (*n*=12). (**B**–**F**) Representative recordings of HeLa cells expressing DRP1 (K38A), showing the effect of different respiratory chain inhibitors on pH_mito_ flashes and on the basal mitochondrial pH. (**G**) Simultaneous mito-SypHer and Rhod-2 recordings. No [Ca^2+^]_mito_ changes were observed during pH_mito_ flashes. (**H**) Effect of Ca^2+^ depletion and Ca^2+^ buffering on the frequency of pH_mito_ flashes. Cells were treated with thapsigargin (Tg) to deplete Ca^2+^ stores and subsequently loaded with BAPTA-AM to chelate cytosolic Ca^2+^ (*n*=26, 21, and 14 cells). (**I**) Effect of mPTP modulators on the frequency of pH_mito_ flashes (*n*=9–44 cells, means±s.e.m.). *P<0.05, **P<0.01, and ***P<0.001. NS, not significant.

cpYFP flash activity has been shown to require an active respiratory chain (Wang et al, 2008; Schwarzlander et al, 2012a). Accordingly, inhibition of complex I, III, IV, and V with rotenone, antimycin, azide, and oligomycin, respectively, decreased the frequency of SypHer flashes (Figure 2A–D). Furthermore, *Rho 0* cells, which lack mito-DNA and thus all H^+^-translocating complexes, lacked pH_mito_ activity (Figure 2A, inset). The strongest inhibitors were antimycin and the protonophore carbonyl cyanide m-chlorophenyl hydrazone (CCCP) (Figure 2A), at doses increasing respiration (Supplementary Figure S2C), which both collapsed ΔpH_m_ (Figure 2C and F). Earlier studies have linked ΔΨ_m_ fluctuations to cytosolic Ca^2+^ elevations (Duchen et al, 1998; Vergun and Reynolds, 2004; Guzman et al, 2010). We could not detect mitochondrial Ca^2+^ elevations with Rhod2 during pH_mito_ flashes (Figure 2G) and neither intracellular Ca^2+^ stores depletion with thapsigargin, cytosolic Ca^2+^ buffering with (1,2-bis(*o*-aminophenoxy) ethane-N,N,N′,N′-tetraacetic acid) aceto-methyl ester (BAPTA-AM) (Figure 2H), nor genetic manipulation of the recently identified mitochondrial H^+^/Ca^2+^ exchanger protein Letm1 (Supplementary Figure S2) had any impact on flash activity. This indicates that pH_mito_ flashes are not driven by mitochondrial Ca^2+^ uptake. ΔΨ_m_ fluctuations were attributed to mPTP opening by ROS (Huser and Blatter, 1999; Jacobson and Duchen, 2002; Wang et al, 2008). In our hands, mPTP inhibitors (cyclosporine A and bongkrekic acid) and ROS scavengers (Tyron and Tocopherol) did not significantly alter pH_mito_ flash frequency (Figure 2I), indicating that flash activity is not driven by mPTP opening, although atractyloside increased flash frequency by ∼5-fold.

### Mito-SypHer is a specific pH indicator insensitive to superoxide

**Figure 3:**
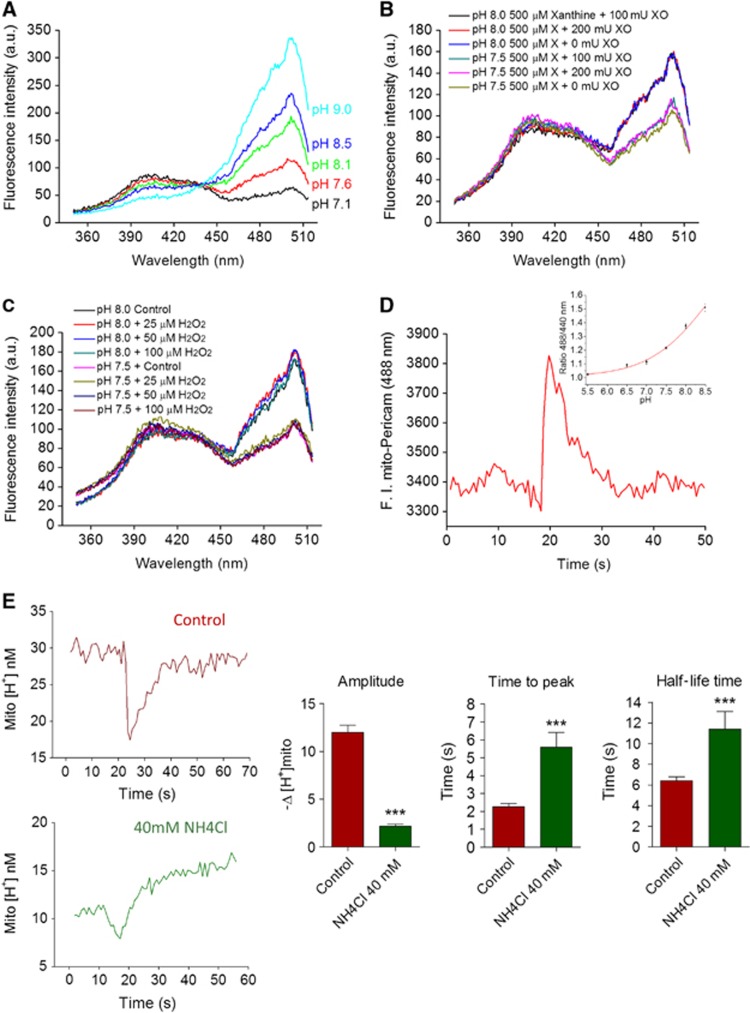
Mito-SypHer is a pH-sensitive probe insensitive to superoxide. (**A**) Excitation spectra (*λ*_em_=530 nm) of purified bacterially expressed SypHer at different pH. (**B**) Excitation spectra of purified SypHer in the presence of xanthine and XO at pH 7.5 and pH 8.0. (**C**) Excitation spectra in the presence of increasing amounts of H_2_O_2_. Spectra are representative of three independent experiments in each condition. (**D**) Spontaneous fluorescence elevations recorded with ratiometric pericam (RP_mit_, *λ*_em_=488 nm) in HeLa cell mitochondria. (*n*=52 transients from three cells). Inset: effect of pH on RP_mit_ F488/F440 ratio fluorescence. (**E**) Effect of 40 mM NH_4_Cl on the kinetics and amplitude of the pH_mito_ elevations recorded with SypHer. Left panels: the fluorescence recordings were converted to proton concentrations and expressed in a non-logarithmic scale to compare the absolute changes in [H^+^]. Right panels: averaged amplitude, time to peak, and half-life time of 56 pH_mito_ elevations recorded in six cells before (red) and after (green) addition of 40 mM NH_4_Cl (means±s.e.m.). ***P<0.001.

The pH_mito_ elevations reported by the ratiometric mito-SypHer probe resemble the superoxide and pH_mito_ flashes previously reported with cpYFP (Wang et al, 2008; Schwarzlander et al, 2012a). To clarify the chemical nature of the flashes, we evaluated the pH and superoxide sensitivity of bacterially expressed 6 × His-tag SypHer. The excitation spectra of purified SypHer was highly sensitive to changes in pH (Figure 3A) but was not affected by the addition of xanthine and xanthine oxidase (XO) at concentrations that evoked a robust superoxide dismutase (SOD)-sensitive increase in the luminescence of the superoxide probe 2-methyl-6-(*p*-methoxyphenyl)-3,7-dihydroimidazo[1,2-alpha]pyrazin-3-one (MCLA) (Figure 3B and Supplementary Figure S3A). Furthermore, the excitation spectra of purified SypHer were not affected by H_2_O_2_ (Figure 3C), by the NO donor *S*-nitroso-*N*-acetyl-DL-penicillamine (SNAP), by the reducing agent dithiothreitol (DTT), or by millimolar concentrations of Ca^2+^, PO_4_^−^, and ATP (Supplementary Figure S3B–E and Poburko et al, 2011). These *in vitro* data indicate that the fluorescence of mito-SypHer is insensitive to changes in redox state, ionic strength, and metabolites, and further validate the probe as a ratiometric pH indicator. Ratiometric pericam targeted to the mitochondria (RP_mit_) reportedly responds to superoxide at 488 nm (Pouvreau, 2010), but this indicator is not specific for superoxide as its fluorescence increases sharply above pH 7.0 (Figure 3D, inset) suggesting that the fluorescence flashes reported by RP_mit_ in HeLa cells (Figure 3D) also reflect an increase in matrix pH. Next, we increased the proton-buffering power of mitochondria with the permeable weak base NH_4_Cl, a procedure expected to alter the kinetics of proton but not of superoxide changes. Since NH_4_Cl increases pH_mito_, the fluorescence data were converted to proton concentrations and expressed in a non-logarithmic scale to compare the absolute changes in [H^+^] (Figure 2E). Both the onset and the recovery of the [H^+^] transients were delayed in the presence of the weak base, and their amplitude decreased by 82% (Figure 3E, *n*=49–59). Thus, increasing the buffering power of mitochondria decreased the amplitude and prolonged the duration of the spontaneous transients, providing independent functional evidence that the SypHer flashes are caused by protons and not by superoxide.

### pH_mito_ flashes are bioenergetic events driven by decreases in ΔΨ_m_

**Figure 4:**
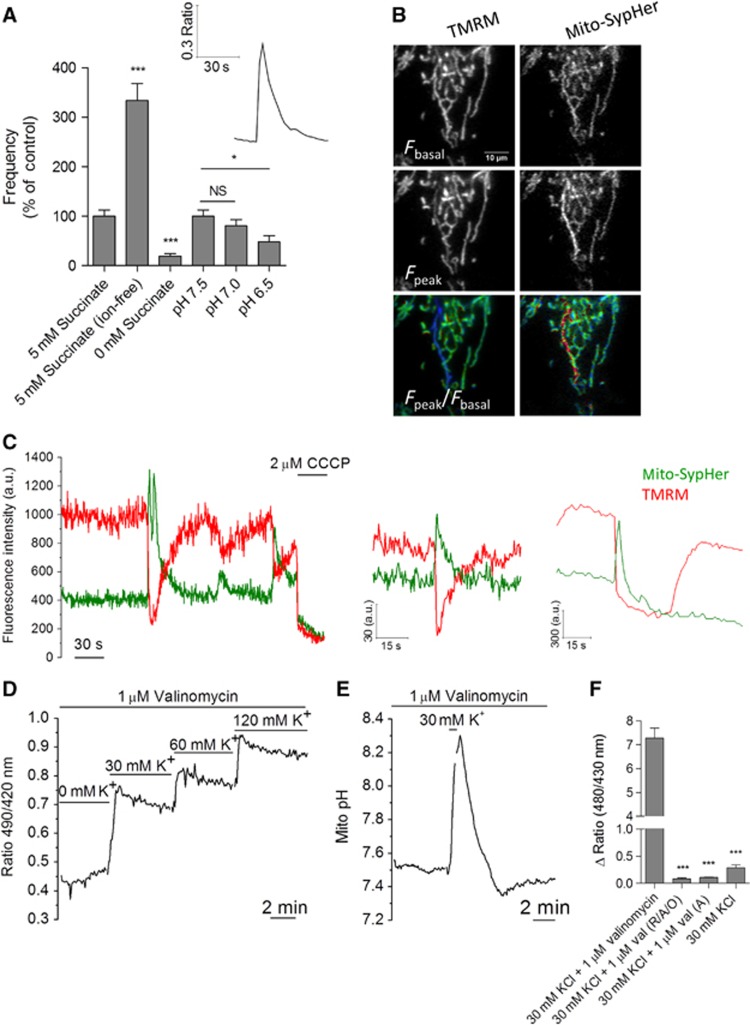
pH_mito_ flashes are bioenergetic events driven by decreases in ΔΨ_m_. (**A**) Spontaneous elevations in mito-SypHer fluorescence in HeLa cells permeabilized in KCl-based solutions. Inset shows a typical pH_mito_ flash, bar graph shows the frequency of flashes recorded in solutions containing (*n*=64) or lacking ions (Ca^2+^, Na^+^, K^+^, and PO_4_^−^ replaced with sucrose, *n*=62), respiratory substrates (*n*=32), and of varying pH (*n*=64 for each condition) (means±s.e.m.). (**B**) Simultaneous pH_mito_ and ΔΨ_m_ recordings in intact cells loaded with TMRM. A concomitant decrease in ΔΨ_m_ was observed in 64/64 flashes. See also Supplementary Movie S2. (**C**) ΔΨ_m_ typically mirrored pH_mito_ (left panels) but occasionally exhibited delayed recovery (right panel). (**D**) Effect of imposed mitochondrial depolarizations on pH_mito_. Permeabilized cells were equilibrated with valinomycin and exposed to increasing concentration of KCl to clamp ΔΨ_m_ at different depolarized potentials. Increasing depolarization steps induced progressive matrix alkalinization (*n*=37 cells). (**E**) pH_mito_ elevation evoked by a brief pulse of 30 mM KCl to transiently depolarize mitochondria (*n*=68). (**F**) Effect of respiratory chain inhibitors and of valinomycin on the amplitude of the pH_mito_ elevation evoked by KCl. A, antimycin; O, oligomycin; R, rotenone; Val, valinomycin (*n*=50 cells for each condition; means±s.e.m.). *P<0.05 and ***P<0.001. NS, not significant.

We next recorded pH_mito_ in cells permeabilized with digitonin and perfused with succinate. pH_mito_ flashes were readily observed in permeabilized cells (Figure 4A, inset), a configuration that, as shown previously (Poburko et al, 2011), allowed basal pH_mito_ levels to vary rapidly and reversibly with the cytosolic pH (Supplementary Figure S4A). Succinate removal reduced flash frequency by 93%, whereas substitution of Na^+^, K^+^, Ca^2+^, Cl^−^, and PO_4_^2−^ with sucrose increased flash frequency without altering their kinetics or amplitude. Decreasing cytosolic pH from 7.5 to 7.0 did not significantly decrease flash frequency, but further acidification to pH 6.5 decreased flash frequency by ∼50% (Figure 4A). The pH_mito_ flashes thus persisted in permeabilized cells, their frequency increasing in ion-free conditions and decreasing under acidic conditions and substrate removal. These data indicate that the pH_mito_ flashes are not driven by the entry of ions into mitochondria, and that the flash activity requires respiring mitochondria and a permissive matrix or cytosolic pH but not cytosolic ions.

The cpYFP flashes occur coincidentally with decreases in ΔΨ_m_ (Wang et al, 2008). We also observed a concomitant decrease in ΔΨ_m_ with every pH_mito_ elevation during simultaneous SypHer and tetramethyl rhodamine methyl ester (TMRM) recordings (Figures 4B–C, *n*=64 events, Supplementary movie S2). In most cases (96%), the depolarization events were transient and mirrored the pH_mito_ elevations (Figure 4C, left traces), but on rare occasions (4%) mitochondria remained depolarized for several seconds after the termination of the pH_mito_ flash (Figure 4C, right traces). The upstroke of the pH_mito_ and ΔΨ_m_ transients was faster than the temporal resolution of our imaging setup (20 Hz), and the two activities thus appeared coincidental. The mirror changes in ΔΨ_m_ and pH_mito_ reflect opposite alterations in the electrical and chemical components of the proton-motive force, suggesting that ΔpH_m_ increases to balance the decrease in ΔΨ_m_ (Santo-Domingo and Demaurex, 2012). To test this possibility, we clamped the ΔΨ_m_ at different potentials by equilibrating permeabilized cells with valinomycin at different K^+^ concentrations. As predicted, addition of 30 mM KCl evoked an immediate increase in pH_mito_, and subsequent additions of higher K^+^ concentrations further alkalinized the matrix (Figure 4D). To test whether a pH_mito_ flash could be evoked by an artificial depolarization, we briefly added 30 mM KCl to cells equilibrated with valinomycin. This treatment faithfully reproduced pH_mito_ flashes, the pH_mito_ rapidly increasing upon KCl addition and slowly recovering upon KCl withdrawal (Figure 4F). KCl had no effect in the absence of valinomycin (Figure 4G) and pH_mito_ flashes were also evoked by addition of NaCl or LiCl to permeabilized cells treated with the ionophore A23187 (Supplementary Figure S4B), ruling out K^+^/H^+^ exchange. The KCl-evoked pH_mito_ elevations were prevented by respiratory chain inhibitors (Figure 4G), confirming that they reflected increases in proton pumping. Thus, pH_mito_ flashes can be artificially generated by an imposed transient mitochondrial depolarization, strongly suggesting that the endogenous flash activity of intact cells reflects compensatory increases in ΔpH driven by spontaneous decreases in ΔΨ_m_. The ΔΨ_m_ changes coincided both spatially and temporally with the matrix pH flashes (Figure 4B and Supplementary Figure S4C), suggesting that the electrical events propagate along connected mitochondria and trigger immediate pH responses in depolarized mitochondria.

### Matrix pH elevations propagate faster than matrix GFP along connected mitochondria

**Figure 5:**
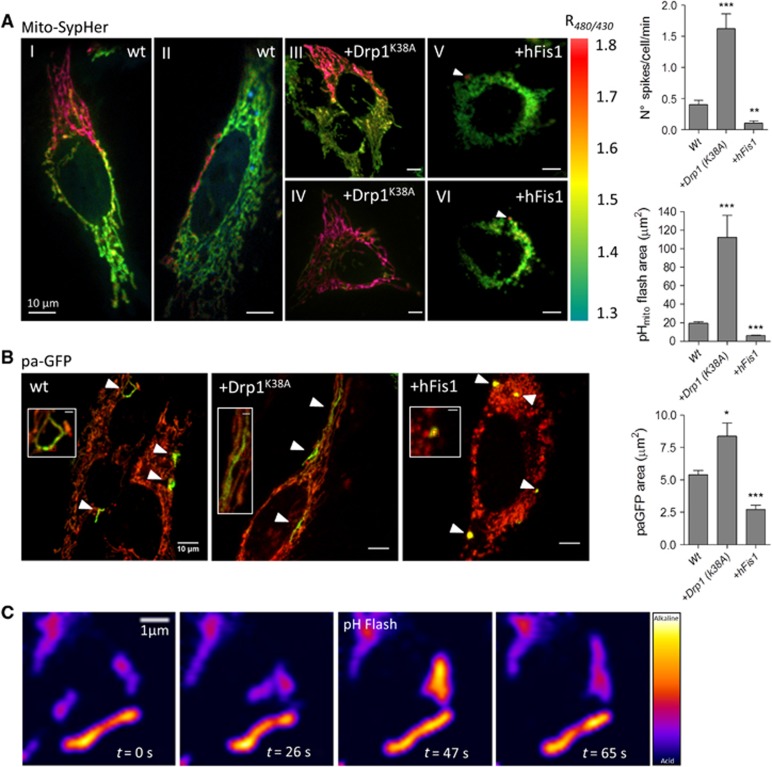
Matrix pH elevations propagate along connected, but not fused, mitochondria. (**A**) Ratio mito-SypHer images taken at the peak of elementary pH_mito_ elevations in WT HeLa cells (I, II) and in cells expressing the pro-fusion protein DRP1^K38A^ (III, IV) or the pro-fission protein hFIS1 (V, VI). Note that in DRP1^K38A^ cells pH_mito_ flashes occasionally spread over the entire mitochondrial network. See also Supplementary Movie S3. Bar graphs: averaged spatial extent and frequency of pH_mito_ flashes in 15 min continuous recordings of 11 cells for each condition (means±s.e.m.). (**B**) Individual matrix compartments labelled with paGFP. Representative merged TMRM/paGFP fluorescence images taken 5 s after paGFP photoactivation in HeLa cells expressing the indicated plasmid; insets show paGFP-labelled regions at higher magnification, with the irradiated region indicated by a crosshair; bar graph: averaged spatial extent of matrix paGFP propagation (*n*=6 independent experiments, means±s.e.m.). (**C**) pH_mito_ flash occurring during transient contact between two individual mitochondria. See also Supplementary Movie S4. *P<0.05, **P<0.01, and ***P<0.001.

The spatial dimension of the pH_mito_ elevations varied considerably, with some events restricted to single mitochondria and other occurring in large clusters of interconnected mitochondria (Figure 5AI). To test whether the area covered by a single pH_mito_ elevation varied with mitochondria interconnectivity, we enforced mitochondrial shape changes by overexpressing mitochondrial-shaping proteins. Elongation was promoted by a dominant-negative DRP1 mutant (DRP1^K38A^) and fission by the pro-fission protein hFIS1 (Frieden et al, 2004). The spatial extension of the pH_mito_ elevations increased dramatically in cells expressing DRP1^K38A^ (Figure 5AIII), with global pH_mito_ flashes observed in some cells (Figure 5AIV and Supplementary movie S3), and decreased in cells expressing hFIS1 (Figure 5AV). On average, a single pH_mito_ flash covered 17.02±0.32 μm^2^ (*n*=81) of the fluorescent mitochondrial area in control HeLa cells, 109.08±21.12 μm^2^ (*n*=96 flashes) in cells expressing DRP1^K38A^, and only 6.41±0.08 μm^2^ (*n*=59) in hFIS1 expressers. Interestingly, the frequency of the pH_mito_ elevations increased in cells with fused mitochondria and decreased in cells with fragmented mitochondria (Figure 5A), while the flash amplitude and time to peak increased upon DRP1^K38A^ and hFIS1 expression, respectively (Supplementary Figure S5A). We next tested whether flash propagation reflected luminal continuity between neighbouring organelles by measuring the matrix diffusion of a photoactivated GFP (paGFP). Photoactivation of matrix-targeted paGFP revealed areas that were much smaller than the areas of elementary pH_mito_ flashes (Figure 5B, the individual matrix compartments labelled with paGFP appearing in green over the red TMRM mitochondrial staining). paGFP fluorescence covered a maximal mitochondrial area 2 s after laser illumination (Supplementary Figure S5B and C), a procedure that did not alter TMRM fluorescence (Figure 5D and E), indicating that our measurements were not limited by the rates of paGFP matrix diffusion or by laser-induced toxicity. paGFP-labelled areas were ∼3-fold smaller than the pH_mito_ flash areas in non-transfected cells and ∼13-fold smaller than flash area in DRP1^K38A^ cells, the paGFP areas increasing by only ∼30% upon DRP1^K38A^ expression while the pH_mito_ flash area increased by ∼6-fold (Figure 5A and B). paGFP-labelled areas of up to 47 μm^2^ could be detected in DRP1^K38A^ expressers, indicating that the small average size of paGFP regions did not reflect failure to detect paGFP fluorescence but rather probe confinement. Photoactivation of matrix-targeted paGFP in cells loaded with TMRM confirmed that the spontaneous ΔΨ_m_ drops propagated within a much wider mitochondrial area than the matrix paGFP (data not shown). These data indicate that pH_mito_ flashes can propagate along interconnected mitochondria that have limited exchange of matrix protein content. Nevertheless, pH_mito_ and ΔΨ_m_ flashes correlated temporally with mitochondrial fusion events in live microscopy (Figure 5C and Supplementary movie S4), indicating that flash activity is linked to mitochondrial fusion.

**Figure 6:**
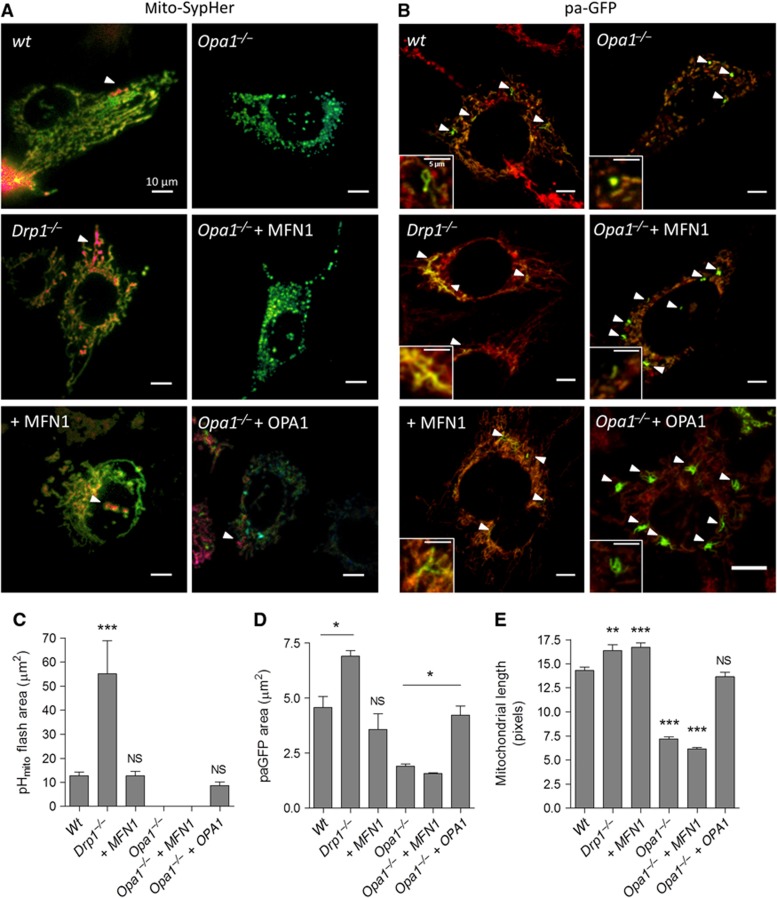
pH_mito_ flashes require OPA1 but not MFN1. (**A**) SypHer images taken at the peak of elementary pH_mito_ elevations in mouse embryonic fibroblasts (MEFs) derived from control, *Drp1*^−/−^, and *Opa1*^−/−^ mice transfected or not with MFN1 or OPA1. (**B**) Matrix compartments labelled with paGFP in cells of the indicated genotype; insets show the paGFP-labelled regions at higher magnification. Averaged spatial extent of (**C**) pH_mito_ flashes and of (**D**) paGFP propagation in 15 min continuous recordings of 30 cells for each condition (*n*=6 independent experiments) (means±s.e.m.). (**E**) Averaged length of individual mitochondria (*n*=300, means±s.e.m.). *P<0.05, **P<0.01, and ***P<0.001. NS, not significant.

To further explore the link between mitochondrial fusion and pH_mito_ flash propagation, we used fibroblasts (MEFs) from knockout mice that completely lack the endogenous pro-fission protein DRP1 (Ishihara et al, 2009) or the inner membrane pro-fusion protein OPA1 (Gomes et al, 2011). The size of pH_mito_ flashes increased by ∼5-fold in *Drp1*^*−/−*^ cells while the area of paGFP regions and the length of the smallest fluorescent objects, an independent readout of mitochondrial length, increased by only ∼30% (Figure 6). *Drp1* ablation thus markedly increased the propagation of pH_mito_ flashes without altering their frequency and amplitude (Supplementary Figure S6), consistent with the phenotype of HeLa cells expressing the dominant-negative DRP1^K38A^. In contrast, expression of MFN1 in WT MEFs increased the length of individual mitochondria as expected but did not increase the size of the pH_mito_ flashes or paGFP areas (Figure 6C–E), indicating that enforced fusion of the outer membrane does not promote pH_mito_ flash propagation. Remarkably, *Opa1* ablation abrogated pH_mito_ flash activity (Figure 6C) and ΔΨ_m_ fluctuations (not shown), and reduced as expected both the length of individual mitochondria and the size of paGFP-labelled areas by half (Figure 6D–E). Although *Opa1*^*−/−*^ are bioenergetically competent (Gomes et al, 2011), not a single pH_mito_ flash was detected in *Opa1*^*−/−*^ cells even after application of atractyloside, which increased pH_mito_ flash frequency by four-fold in control cells (Supplementary Figure S6E), or after expression of MFN1, which, as expected, Cipolat et al (2004) failed to rescue mitochondrial length and paGFP propagation (Figure 6). Importantly, re-expression of OPA1 restored pH_mito_ flash activity, mitochondrial length, and paGFP matrix propagation to WT levels (Figure 6). These data indicate that OPA1-mediated fusion of the inner membrane, but not MFN1-mediated fusion of the outer membrane, is linked to the pH_mito_ flash activity.

**Figure 7:**
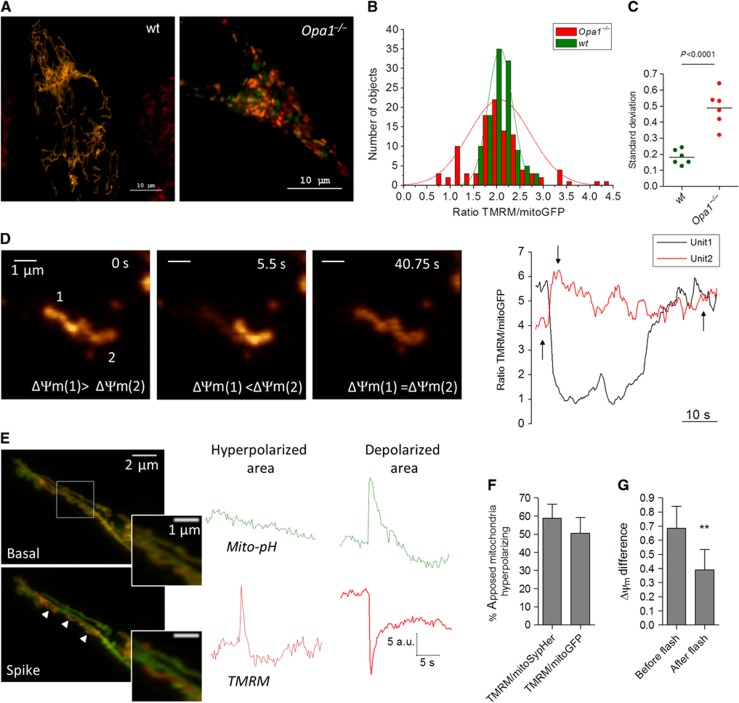
OPA1-mediated pH_mito_ flashes equilibrate the potentials of apposed mitochondria. (**A**) Representative images of wt and *Opa1*^−/−^ MEFs expressing matrix GFP and loaded with TMRM. (**B**) Distribution of ΔΨ_m_ (expressed as TMRM/mitoGFP ratio of 2.5-μm^2^-wide fluorescent objects) from these two cells. (**C**) S.d.’s of ΔΨ_m_ Gaussian distributions in mitochondrial populations from six wt and six *Opa1*^−/−^ cells. (**D**) Concomittant hyperpolarization (#1) and depolarization (#2) events in two adjacent mitochondria. Note that the two mitochondrial potentials equilibrated after the event. See Supplementary Movies S5a and S6b. (**E**) ΔΨ_m_ and pH_mito_ recordings of adjacent mitochondria undergoing opposite changes in ΔΨ_m_. The pH_mito_ remained stable in the hyperpolarizing mitochondrion. (**F**) Fraction of adjacent mitochondria (defined as closer than 2 pixels from a flashing mitochondrion) undergoing concomitant hyperpolarization events (*n*=20–29 cells). (**G**) ΔΨ_m_ difference between adjacent mitochondria measured before and after the synchronous hyperpolarizing/depolarizing event (*n*=23 events, means±s.e.m.). **P<0.01.

We next tested the impact of OPA1-mediated flash activity on mitochondrial bioenergetics, using ratiometric imaging of TMRM over matrix-targeted GFP to quantify ΔΨ_m_. *Opa1* ablation markedly increased the heterogeneity of ΔΨ_m_ within the mitochondrial population of individual cells (Figure 7A). The ΔΨ_m_ of 2.5-μm^2^-wide fluorescent objects had a Gaussian distribution (Figure 7B) whose s.d. increased by 2.5-fold upon *Opa1* ablation, from 10 to 25% (Figure 7C). This indicates that OPA1-mediated fusion promotes the equilibration of mitochondrial membrane potentials. We therefore checked whether this ΔΨ_m_ equilibration was linked to the pH_mito_ flash activity. The distribution of ΔΨ_m_ (measured with TMRM or TMRM/GFP) and of pH_mito_ (measured with SypHer) remained unchanged within the flashing regions (Supplementary Figure S7), indicating that pH_mito_ flashes do not promote energy equilibration within the flashing regions themselves. However, careful analysis revealed that 66% of flashes were associated with hyperpolarization events occurring in adjacent mitochondria (located <2 pixels from a flashing unit). The ΔΨ_m_ changes occurring in these adjacent mitochondria were synchronous but of opposite direction (Figure 7D and Supplementary Movie S5). The hyperpolarization events were not associated with changes in pH_mito_ (Figure 7E) and occurred in ∼50% of mitochondria adjacent to a flashing unit (Figure 7F) but never in non-adjacent mitochondria. Importantly, the membrane potentials of the two adjacent mitochondria equilibrated after the event (Figure 7D). On average, the ΔΨ_m_ difference between adjacent mitochondria undergoing opposite changes in membrane potential decreased by ∼40% after a flash (Figure 7G). These data show that OPA1-dependent flashes equilibrate the membrane potentials of apposed mitochondria.

## Discussion

In this study, we provide several new insights into the mechanism and significance of spontaneous mitochondrial fluctuations. First, we clarify the chemical nature of ‘mitochondrial flashes’ by using a probe that we show to be responsive to pH but not to superoxide. Superoxide flashes coinciding with ΔΨ_m_ decreases were reported in individual mitochondria from skeletal muscle and intact beating hearts (Wang et al, 2008; Pouvreau, 2010; Fang et al, 2011; Wei et al, 2011), but the cpYFP probe used was shown to be highly sensitive to pH (Schwarzlander et al, 2011) and was subsequently used to report matrix alkalinization transients in mitochondria from *Arabidopsis thaliana* root cells (Schwarzlander et al, 2012a). The pH/superoxide flashes coincide with ΔΨ_m_ decreases and have similar kinetics and pharmacological profiles, suggesting that they reflect the same bioenergetic event. However, since the two activities were measured with the same cpYFP-based probe reportedly sensitive to both proton and superoxide, the chemical nature of the measured signal is uncertain. By showing that our SypHer probe is insensitive to superoxide, we demonstrate that human mitochondria do in fact transiently alkalinize during decreases in ΔΨ_m_, validating the results obtained in astrocytes (Azarias and Chatton, 2011) and plants (Schwarzlander et al, 2012a). The SypHer flashes were altered by increased mitochondrial buffering but not by ROS scavengers and were not associated with fluorescence changes of redox-sensitive green fluorescent protein (roGFP) (data not shown), consistent with protons and not superoxide as the source of the signal. Furthermore, we show that the most potent flash inhibitors, antimycin and CCCP, which are not expected to abrogate superoxide flashes as they increase superoxide production (Muller, 2009), both collapsed ΔpH_m_ as expected from their pharmacology. Since our probe is insensitive to superoxide, we cannot rule out that superoxide flashes occur concomitantly with pH_mito_ flashes, and this possibility should be evaluated with new probes selective for superoxide and insensitive to pH.

Second, we have causally linked the pH_mito_ flashes to the ΔΨ_m_ decreases by demonstrating that prototypical pH_mito_ flashes can be evoked by artificial depolarization of mitochondria with valinomycin/K^+^. In isolated mitochondria equilibrated with valinomycin/K^+^, alterations in ΔΨ_m_ are exactly balanced by opposite alterations in ΔpH, and Δ*p* remains constant within a wide range of voltages (Nicholls, 1974; Lambert and Brand, 2004; Nicholls, 2005). We show here that this rule holds true also in permeabilized cells. A transient mitochondrial depolarization thermodynamically favours H^+^ extrusion by decreasing the driving force for proton pumping by respiratory chain complexes, increasing the rate of H^+^ extrusion and of O_2_ consumption by mitochondria (Nicholls, 1974; Costa et al, 1984; Nicolli et al, 1991; Talbot et al, 2007). The demonstration that this compensatory mechanism occurs spontaneously in intact cells indicates that ΔΨ_m_ fluctuations are not an indicator of mitochondrial dysfunction as concomitant pH_mito_ flashes preserve the proton-motive force, thus maintaining the ability of mitochondria to convert energy. A thermodynamically similar mechanism was recently proposed to account for coincident ΔΨ_m_ and pH_mito_ fluctuations in plant mitochondria, where the pulsing activity was proposed to be triggered by the entry of calcium ions into the matrix (Schwarzlander et al, 2012a). In our hands, mitochondrial Ca^2+^ uptake does not appear to trigger pH_mito_ elevations because the spontaneous activity was not altered by Ca^2+^ store depletion, by cytosolic Ca^2+^ chelation (Figure 2), or by knockdown of the mitochondrial H^+^/Ca^2+^ exchanger Letm1 (Supplementary Figure S2). By comparing the ΔΨ_m_ and pH_mito_ changes recorded during flashes to the changes evoked by CCCP and oligomycin, we can estimate that ΔΨ_m_ was around −120 mV at rest and decreased to −50 mV during a flash, while pH_mito_ averaged 7.8 at rest and increased by 0.4 pH unit during a flash. The IMS pH was previously measured at 6.8 in HeLa cells (Porcelli et al, 2005), and since this parameter strongly depends on proton pumping across the IMS, an acidification of 0.4 pH unit that would match the matrix alkalinization during a flash seems reasonable. We therefore estimate that ΔpH_m_ is around 1 (−60 mV) at rest and increases to 1.8 (−110 mV) during a flash. Based on these calculations, the resting proton-motive force of −180 mV decreases by only ∼20 mV during a flash, but the relative contributions of its electrical and chemical components become inverted.

**Figure 8:**
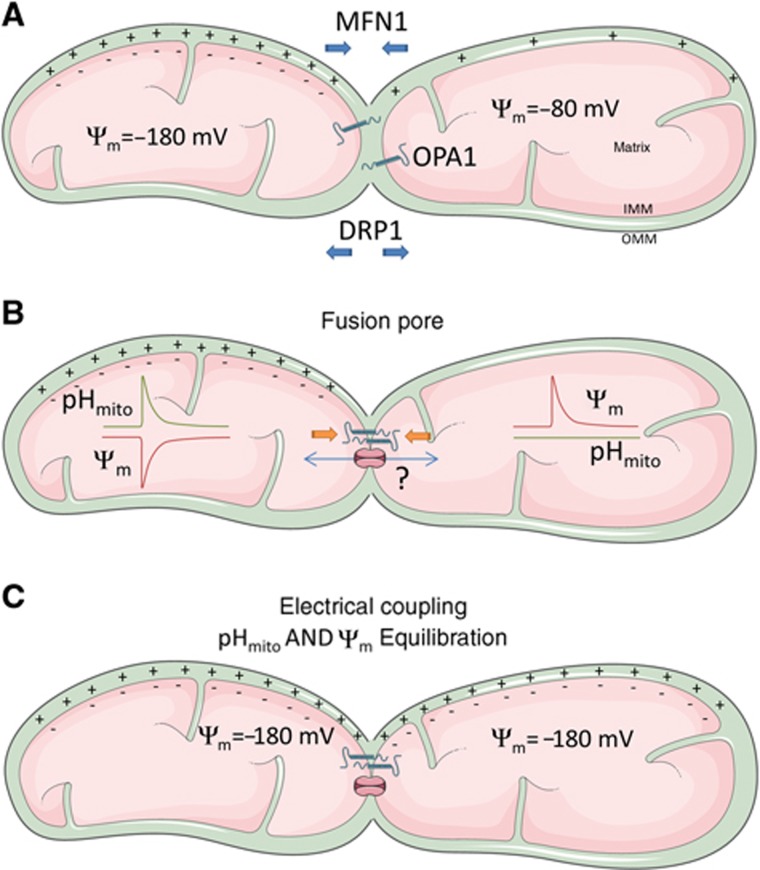
Proposed mechanism of mitochondrial ΔΨ_m_/pH_mito_ fluctuations. We propose that the ΔΨ_m_/pH_mito_ fluctuations are triggered by the opening of a fusion pore between two mitochondria of different potential. (**A**) Individual mitochondria can maintain different ΔΨ_m_ and pH_*m*_. (**B**) OPA1-mediated inner membrane fusion leads to the formation of a fusion pore that establishes electrical continuity between adjacent mitochondria, without allowing the diffusion of matrix proteins. The disequilibrium in membrane potentials drags electrons from the more energized mitochondria, causing a decrease in ΔΨ_m_ that boosts proton pumping by the respiratory chain, generating a pH_mito_ flash in the depolarizing mitochondria. Electrical coupling causes the apposed mitochondria to hyperpolarize during the flash, shutting down proton pumping and preventing pH_mito_ flash propagation in the hyperpolarizing mitochondria. (**C**) At the end of the flash, the two interconnected mitochondria are in electrochemical equilibrium.

Third, we show that OPA1-mediated IMM fusion is required for the generation of the coupled pH_mito_/ΔΨ_m_ fluctuations. ΔΨ_m_ flickers or oscillations were previously linked to mitochondrial Ca^2+^ or Na^+^ entry (Duchen et al, 1998; De Giorgi et al, 2000; Buckman and Reynolds, 2001; Jacobson and Duchen, 2002; Vergun et al, 2003; Vergun and Reynolds, 2004; Azarias et al, 2008) or attributed to mPTP opening by mitochondria ROS (Huser and Blatter, 1999; De Giorgi et al, 2000; Jacobson and Duchen, 2002). We show that pH_mito_ flashes persist in ion-free solutions and are not affected by mPTP inhibitors and ROS scavengers, ruling out cation entry across the mPTP as a trigger of the fluctuations. The unexpected and opposite effects of atractyloside and oligomycin might reflect the diverging effects of these inhibitors on the fusion process. Oligomycin inhibits the ATP synthase while atractyloside inhibits the ANT (Vergun and Reynolds, 2004), causing opposite changes in matrix ATP levels that might differently modulate the formation of a fusion pore. Our observation that the activity disappears in mitochondria lacking the pro-fusion protein OPA1 indicates that the fluctuations are probably linked to the fusion of the mitochondria inner membranes, but this activity differs from the ‘kiss-and-run’ mode of transient mitochondrial fusion previously reported (Liu et al, 2009), which allowed exchange of soluble matrix proteins and promoted mitochondrial mobility. Instead, we propose that the coupled pH_mito_/ΔΨ_m_ fluctuations reflect the transient openings of a fusion pore between contiguous mitochondria of different membrane potentials (Figure 8). Opening a fusion pore will establish electrical continuity between these mitochondria, decreasing ΔΨ_m_ in the more energized mitochondria. The ΔΨ_m_ decrease will then boost proton pumping by active respiratory chain complexes, generating a pH_mito_ flash in the depolarizing mitochondria. Electrical coupling causes the apposed mitochondria to hyperpolarize during the flash, shutting down proton pumping and preventing pH_mito_ flash propagation in the hyperpolarizing mitochondria. While the existence of pores electrically coupling mitochondria awaits electrophysiological confirmation, our ‘junctional coupling’ model explains why flash activity is not coupled to ion fluxes and why mitochondria can preserve their bioenergetic competence during the flashes, as connecting two matrixes will not dissipate the proton-motive force. Previous models have implicated the opening of ion channels, transporters, or large conductance pores between the matrix and the IMS/cytosol, processes that have profound impacts on mitochondria bioenergetics and ionic homeostasis. By linking flash activity to OPA1-mediated mitochondrial fusion, a highly regulated cellular process sensitive to calcium elevations and to oxidative stress (Cereghetti et al, 2008; Tang et al, 2009), our model also accounts for the reported effects of calcium and ROS on flashing activity.

Finally, our concurrent paGFP and TMRM recordings demonstrate that contiguous mitochondria can synchronize their energetic state without mixing their matrix content. Earlier studies had shown that ΔΨ_m_ flickering could propagate along interconnected mitochondria (De Giorgi et al, 2000), but whether ΔΨ_m_ propagation required matrix continuity was not known. Here, we show that inhibition of endogenous DRP1 activity either by genetic ablation or by expression of a dominant-negative mutant greatly enhances the propagation of pH_mito_ elevations along interconnected mitochondria, but marginally increases the propagation of a photoactivated matrix protein. Mitochondrial fusion proceeds unabated in DRP1 incapacitated cells. However, although these mitochondria appear fused on the confocal microscope, their matrix compartments do not allow the free diffusion of matrix paGFP. In contrast, the pH_mito_ fluctuations covered on average 50% of the mitochondrial area and became global in some cells, in which the matrix pH of the whole network increased within 10 ms without any clear initiation spot or visible decay in flash amplitude along labelled structures (Supplementary movie S3). This indicates that the coincident ΔΨ_m_/pH_mito_ fluctuations propagate by a saltatory mechanism or by a very fast regenerative mechanism. Our observation that ΔΨ_m_ and pH_mito_ rapidly equilibrate along interconnected mitochondria confirms the hypothesis originally formulated by Zorov that ΔΨ_m_ and ΔpH_m_ immediately spread along mitochondrial inner membranes (Amchenkova et al, 1988). We extend this concept by showing that the proton-motive force can equilibrate within milliseconds in contiguous mitochondria that do not mix their matrix protein content. One purpose of mitochondrial fusion is to allow genetic complementation of damaged mitochondrial DNA, but this function requires the mixing of matrix content. We demonstrate that another function of mitochondrial fusion proteins is to electrically couple individual mitochondria in order to synergize their metabolic activity.

In summary, we show here that mitochondria exhibit spontaneous elevations in their matrix pH triggered by bursts of depolarization that both propagate faster than matrix GFP along connected mitochondria. This indicates that mitochondria can be electrically coupled without exchanging their matrix content. We propose that the matrix alkalinization transients reflect increased pumping by the respiratory chain during ΔΨ_m_ decreases triggered by the opening of a fusion pore between neighbouring mitochondria of different membrane potentials. This new mode of mitochondrial coupling might facilitate the transmission of energy inside cells by equilibrating the proton-motive force along electrically connected, but not fused, mitochondria.

## Materials and methods

### Cell culture and transfection

HeLa cells and 143b cells (*wt* and *Rho 0*) were cultured in Dulbecco’s modified Eagle’s medium (DMEM) with 1 and 4.5 mg/ml glucose, respectively; WT and *Drp1*^*−/−*^ MEF cells in DMEM-Glutamax with non-essential amino acids; *Opa1*^*−/−*^ cells in MEF medium supplemented with uridine (50 μg/ml, Sigma). All media contained 10% FCS, 1% penicillin, and 1% streptomycin. For fluorescence imaging, 10^5^ cells were seeded on 25-mm glass cover slips, transfected 24 h later with 2 μg of DNA and 5 μl Lipofectamine 2000, and imaged 48 h later. All reagents were purchased from Invitrogen or Sigma.

### Mito-SypHer purification and *in vitro* characterization

The N-terminal poly-His-tag mito-SypHer was generated by cloning in frame mito-SypHer into the Xho-I/ Hind-III site of the prokaryotes expression vector pBAD/HisB (Invitrogen). TOP10-competent cells (Invitrogen) were transformed and expression induced according to the manufacturer’s instructions in the presence of 0.002% arabiniose for 4 h. Cells were lysed and total protein content kept in Tris 25 mM, 150 mM NaCl. After purification in a nickel column, the samples were dialysed at 4°C for 16 h and mito-SypHer concentrated using Amicon Ultra-4 Centrifugal Filter Units (Millipore). Five millimolar β-mercaptoethanol was present throughout the isolation procedures to reduce thiol groups. Twenty microlitre (2 μM) of purified mito-SypHer was dissolved in 200 μl Tris 25 mM, 150 mM NaCl pH 7.5 (0.5 mM β-mercaptoethanol), and fluorescence spectra recorded on a LS50B spectrometer (Perkin Elmer), using 500 μM xanthine and 100 mU XO (Sigma) to generate superoxide.

### Superoxide production measurements *in vitro*

Xanthine (X)/XO O_2_^−^ production was verified by adding 0.01 mM of the luciferin analogue MCLA to a buffer containing 25 mM Tris, 150 mM NaCl, 0.3 mM ethylenediaminetetraacetic acid (EDTA), 0.3 mM β-mercaptoethanol, and 400 μM xanthine (pH 7.5). XO (100 mU) was added to initiate the reaction and the luminescence recorded every 10 s on a FLUOstar (BMG Labtech) microplate reader. To verify the O_2_^−^ specificity of the signal emitted by MCLA, 50 U/ml SOD was included in control experiments.

### Mitochondrial pH, ΔΨ_m_, and Ca^2+^ measurements in live cells

Recordings were performed in N-2-hydroxyethylpiperazine-N′-2-ethane sulphonic acid (HEPES) buffer solution containing 140 mM NaCl, 5 mM KCl, 1 mM MgCl_2_, 2 mM CaCl_2_, 20 mM HEPES, 10 mM glucose, pH set to 7.4 with NaOH at 37°C. The Ca^2+^-free solution contained 0.5 mM ethyleneglycol-bis (beta-aminoethylether)-*N*,*N*′-tetraacetic acid (EGTA) and no CaCl_2_. Mito-SypHer was alternately excited for 200–300 ms at 440 and 488 nm on a Nipkow spinning disk confocal microscope (Visitron Systems GmbH) equipped with a × 63 1.4 NA oil-immersion objective (Carl Zeiss AG). Images were acquired every 800 ms and ratios calculated in MetaFluor 6.3 (Universal Imaging) and analysed in Excel (Microsoft) and GraphPad Prism 5.01 (GraphPad Inc, La Jolla, USA). Mitochondrial pH was calibrated using nigericin (5 μg/ml) and monensin (5 μM) in 125 mM KCl, 20 mM NaCl, 0.5 mM MgCl_2_, 0.2 mM EGTA, and, Tris (pH 8.0, 9.0), HEPES (pH 7.0–7.5), or MES (pH 5.5–6.5). For each cell, a 5-point calibration curve was fitted to a variable slope sigmoid equation with 1/*y* weighting and constraining the top of the curve to 30 (GraphPad Prism 5.01). For simultaneous pH_mito_/Ca^2+^_mito_ measurements, cells were incubated at room temperature for 30 min with 2 μM Rhod-2-AM, washed for 20 min, and imaged immediately. For pH_mito_/Ψ_*m*_ recordings, cells were incubated at room temperature for 20 min with 4 nM TMRM, washed, and kept at 37°C on the microscope until signal reached stability. Mito-SypHer was excited for 300 ms at 488 nm and TMRM or Rhod-2 were excited for 300 ms at 565 nm. Image pairs were acquired every 600 ms.

### Measurements in permeabilized cells

Cells were imaged with a × 40, 1.3 NA objective (Zeiss Axiovert s100TV) using a cooled CCD camera (MicroMax, Roper Scientific). For pH imaging, SypHer was alternately excited for 200–300 ms at 430 and 480 nm through a 505DCXR dichroic filter and imaged with a 535DF25 band pass filter (Omega Optical). Cells were permeabilized by a short exposure to digitonin (1 min, 100 μM) in a buffer containing 120 mM KCl, 10 mM NaCl, 1 mM H_2_KPO_4_, 20 mM HEPES, 5 mM succinic acid, 1 mM ATP-Mg^2+^, 0.02 mM ADP-K, 1 mM MgCl_2_, 0.5 mM EGTA adjusted to pH 7.4 with KOH. The ion-free solution contained 10 mM HEPES, 5 mM succinic acid, 0.5 mM EGTA, and sucrose to reach 300 mOsm at pH 7.4. For manipulations of the mitochondrial membrane potential, 1 μM valinomycin was added and sucrose and KCl balanced to reach 300 mOsm at pH 7.4.

### Mitochondrial length analysis

Confocal Z-stacks of cells expressing matrix-targeted red fluorescent protein (mtRFP) were acquired on a Nikon 1 AR inverted Microscope using a × 60 objective (oil; CFI Plan APO 1.4 NA) and 561 nm (50 mW) excitation. Analysis of mitochondrial length was performed with Image J tool ‘Freehand line selection’ by measuring 10 mitochondria per cell (>30 cells per condition; three experiments).

### paGFP experiments

Cells transiently expressing paGFP were loaded for 25 min at 37°C with 4 nM TMRM in imaging buffer (135 mM NaCl, 5 mM KCl, 0.4 mM KH_2_PO_4_, 1 mM MgSO_4_*7H_2_O, 20 mM HEPES, 0.1% D-glucose, pH 7.4). Cells with low GFP fluorescence intensity were selected to avoid saturation of the GFP emission upon photoactivation (Patterson and Lippincott-Schwartz, 2002). GFP and TMRM were imaged concurrently on the confocal microscope with the objective described above, using 488 and 561 nm excitation and 520/35 and 624/40 emission filters, respectively. Four images were acquired (one image/second) before applying one single stimulation pulse (500 ms, 405 nm laser, 100% power) followed by live imaging at 1 frame/second for 1 min. Loss of focus and movement artifacts was minimized by using a large pinhole aperture (6.9 AU), the Perfect Focus system (Nikon), and by checking photoactivated areas in ratio images. The NIS Elements AR3.2 software was used for data acquisition and analysis of the area of paGFP detected with ‘Auto Detect Area’. Photoactivated areas were measured 5 s after photoactivation to allow paGFP equilibration across the lumen of the mitochondrial network (Twig et al, 2006).

### Rapid pH and potential measurements

Time-resolved pH and potential imaging was performed on cells transiently transfected with mito-SypHer and loaded with TMRM, using the IMIC Andromeda system (Fondis Electronic) equipped with a × 60 oil objective (UPLAN × 60 oil, 1.35NA, Olympus), 488 and 561 nm lasers for excitation and FF01-446/523/600/677 (Semrock) as emission filter. Two or four binning and cropped sensor mode was used to increase frame rate. The two images were acquired with the same exposure time of 15 ms to obtain acquisition rates of 66 frames per second.

### Statistical analysis

All statistical analyses were performed using Prism software (GraphPad). Significance between two sets of experiments was determined using a Student’s *t*-test whereas group sets were analysed using ANOVA.

## Supplementary Material

Supplementary Movie S1

Supplementary Movie S2

Supplementary Movie S3

Supplementary Movie S4

Supplementary Movie S5a

Supplementary Movie S5b

Supplementary Movie Legends

Supplementary Information

Review Process File
