# Supplementary material for: OPA1 promotes pH flashes that spread between contiguous mitochondria without matrix protein exchange
Source: EMBO J. 2013 May 28;32(13):1927–40. doi: 10.1038/emboj.2013.124 (PMC3981180; doi:10.1038/emboj.2013.124)
Supplement: Supplementary Movie Legends [file emboj2013124s7.docx]

**Movie S1 (related to Figure 1A). Transient matrix pH elevations in mitochondrial clusters.** Time-lapse sequence of F480/F430 ratio fluorescence changes in a HeLa cell expressing mito-SypHer. Spontaneous alkalinisation transients occur repeatedly in individual mitochondrial clusters. Green/yellow colours denote high ratio values.

**Movie S2 (related to Figure 4B). Simultaneous pH_mito_ and ΔΨ_m_ recordings.** Time-lapse sequence of mito-SypHer (left window, ex: 488 nm) and TMRM (right window, ex: 565 nm) fluorescence changes in a single HeLa cell. A transient mitochondrial alkalinization concomitant with a drop in membrane potential occurs in the long mitochondrial structure at left.

**Movie S3 (related to Figure 5A). Global matrix pH flashes in cells with fused mitochondria.** Time-lapse sequence of mito-SypHer F480/F430 ratio fluorescence changes in a Hela cell expressing the pro-fusion protein DRP1^K38A^. The spontaneous mitochondrial pH flashes spread over the entire mitochondrial network.

**Movie S4 (related to Figure 5C). Temporal correlation between mitochondrial fusion and pH flashes.** Time-lapse sequence of mito-SypHer (green) and TMRM (red) fluorescence changes during transient contact between two individual mitochondria. A spontaneous pH flash (appearing green in theses overlaid images) occurred after mitochondrial contact.

**Movie S5a (related to Figure 7D). Mitochondrial membrane potential equilibration after a flash-Ratio.** Time-lapse sequence of TMRM over mitoGFP ratio fluorescence changes in two adjacent mitochondrial units undergoing simultaneous and opposite changes in membrane potential. Note that the membrane potentials of the two mitochondria differed before and equilibrated after the transient event.

**Movie S5b (related to Figure 7D). Mitochondrial membrane potential equilibration after a flash-Gold.** Time-lapse sequence of the TMRM fluorescence changes (colour-coded in Gold) in the two mitochondria shown in movie S5a.
