# Supplementary material for: OPA1 promotes pH flashes that spread between contiguous mitochondria without matrix protein exchange
Source: EMBO J. 2013 May 28;32(13):1927–40. doi: 10.1038/emboj.2013.124 (PMC3981180; doi:10.1038/emboj.2013.124)
Supplement: Supplementary Information [file emboj2013124s8.pdf]

SUPPLEMENTARY FIGURE LEGENDS

Figure S1 (related to Figure 1) *In situ* mito-sypHer pH titration. Hela cells expressing mito-SypHer were sequentially bathed with a high K^+ solution containing 5 μg/ml Nigericin at pH 5.5, 6.5, 7.0, 7.5 and 8.0. Average of 5 independent experiments (34 cells, means ±SEM).

Figure S2 (related to Figure 2) Effect of Letm1 levels on pH flash frequency. (A) Letm1 mRNA levels quantified by real time PCR, data normalized by the mRNA averaged levels of 3 housekeeping genes. (B) Frequency of pH flashes in Letm1 siRNA transfected cells compared with control. Data obtained by the analysis of cell frequency in 10 different coverslips per experimental condition. (C) Effect of 2 μM CCCP on oxygen consumption by HeLa cells, measured with a Clark electrode.

Figure S3 (related to Figure 3) In vitro superoxide generation and SypHer properties. (A) Superoxide generation by xantine/xanthine oxidase. O_2^- production was measured with MCLA in the buffer used to record SypHer spectra (pH 7.5). Average of 3 independent experiments (means ±SEM). Addition of 100 mU xanthine oxidase caused a transient increase in luminescence that was completely prevented by superoxide dismutase (SOD, red trace). Representative of 3 independent experiments for each condition. (B-E) Excitation spectra of purified SypHer (λ_{em}=530nm) recorded at pH 7.5 in the presence of increasing amounts of SNAP, DTT, $CaCl_2$, and ATP. Data are representative of 3 independent experiments for each condition.

Figure S4 (related to Figure 4). pH_{mito} flashes are driven by concomitant decreases in $\Delta\Psi_m$. (A) HeLa cells expressing mito-SypHer were permeabilized with 100 μM digitonin for 1 min in KCl-based solutions, and the bath pH was then varied from pH 7.5 to 7.0 and 6.5. Traces are representative recordings from 6 different cells. (B) pH_{mito} elevations evoked by short pulses of 30mM NaCl and 30mM LiCl. Permeabilized cells expressing mito-SypHer were equilibrated with 5μM A23187 and exposed to brief pulses of 30mM NaCl and 30mM LiCl to induce a transient mitochondrial depolarization (n=29 cells). (C) Spatiotemporal coincidence of $\Delta\Psi_m$ and pH_{mito} fluctuations. Simultaneous recordings at 28 frames per second of mito-SypHer and TMRM in HeLa cells. The fluorescence traces correspond to the region of interest zoomed out in the upper panel.

Figure S5 (related to Figure 5). Effects of DRP1^{K38A} and hFIS1on pH$_{mito}$ flash kinetics and paGFP matrix diffusion. (A) Averaged time to peak, half-life time, and amplitude of the pH$_{mito}$ flashes shown in Figure 5A. (n=90, means ±SEM). (B) paGFP matrix diffusion after photoactivation. Images show merged mito-RFP/paGFP fluorescence at different times after paGFP photoactivation. (C) Kinetics of paGFP spatial propagation following photoactivation in 40 different mitochondrial areas. (D) TMRM and paGFP fluorescence intensities before and immediately after photoactivation in HeLa cells expressing mito-paGFP and loaded with 4nM TMRM. (n=25, means ±SEM). (E) TMRM fluorescence intensity within irradiated area before and after laser illumination in Hela cells loaded with 4nM TMRM. (n=74, means ±SEM).

Figure S6 (related to Figure 6). Effects of *Opa1* and *Drp1* ablation on pH$_{mito}$ flash properties. (A-D) Averaged frequency, amplitude, and kinetics of the pH$_{mito}$ flashes shown in Figure 6 (n=50 events). (E) Effect of 20 µM Atractyloside on the pH$_{mito}$ flashes recorded in wt and in *Opa1*$^{-/-}$ MEFs cells. (n=50 events; means ± SEM).

Figure S7 (related to Figure 7). Effect of pH$_{mito}$ flashes on $\Delta\Psi_m$ and pH$_{mito}$ heterogeneity. (A) Standard deviation of TMRM and SypHer fluorescence intensities measured within flashing regions before and after $\Delta\Psi_m$/pH$_{mito}$ flashes (n=32 events) (B) Standard deviations of TMRM/mitoGFP ratio fluorescence measured within flashing regions before and after flashes. (n=20 events; means ± SEM).

S.1

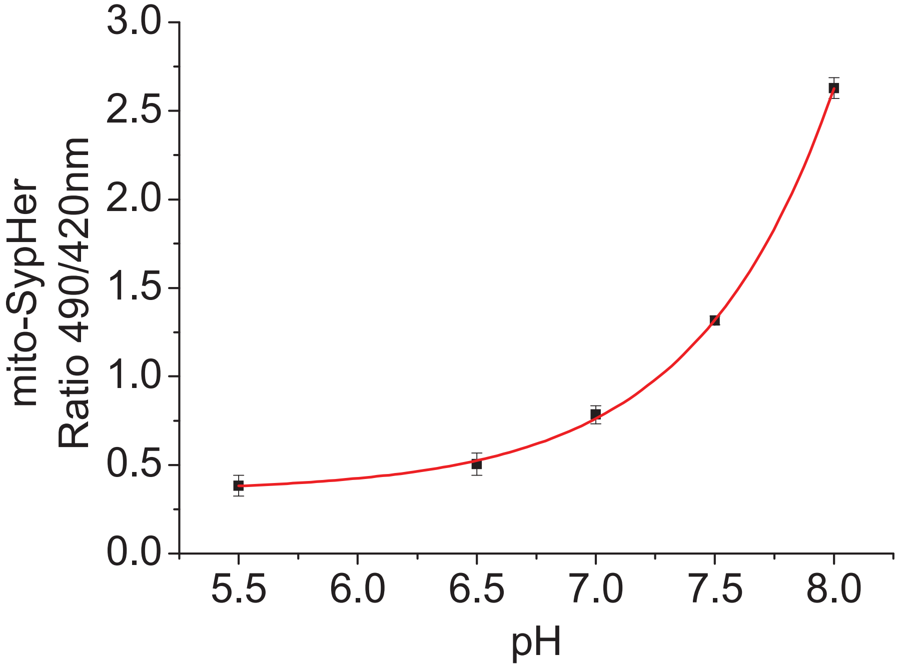
mito-SypHer
Ratio 490/420nm
3.0
2.5
2.0
1.5
1.0
0.5
0.0
5.5
6.0
6.5
7.0
7.5
8.0
pH

S.2

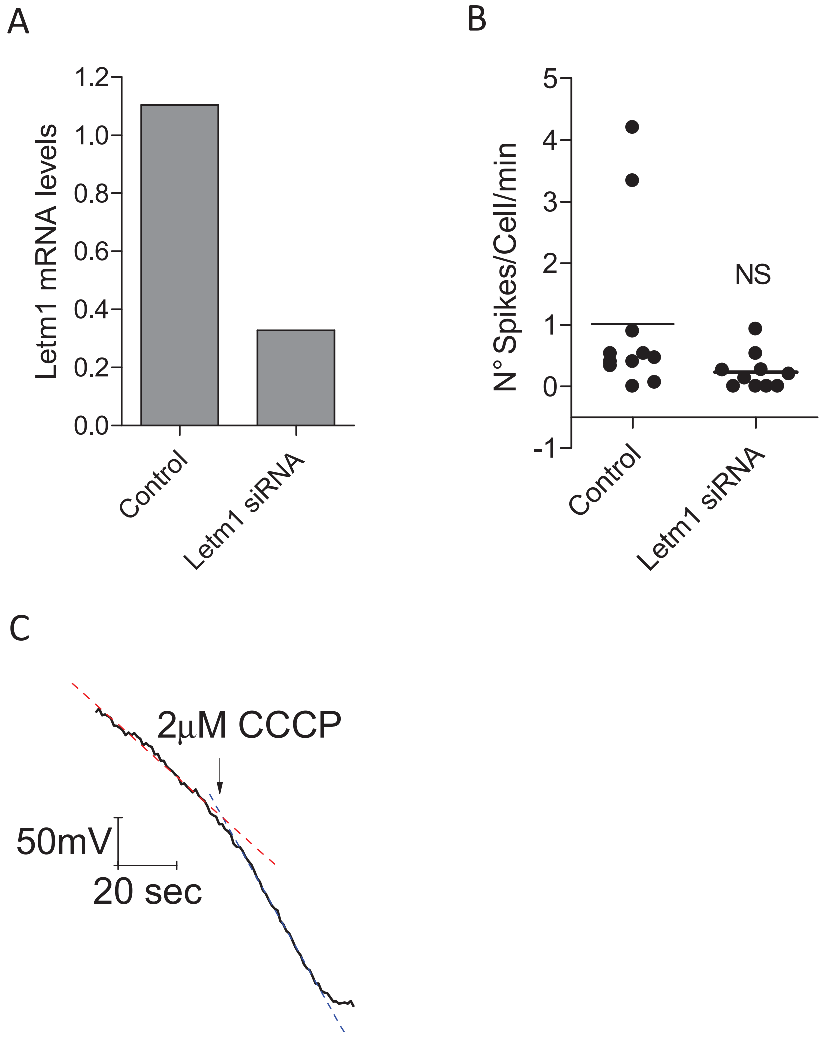
A
1.2
1.0
0.8
0.6
0.4
0.2
0.0
Letm1 mRNA levels
Control
Letm1 siRNA
B
5
4
3
2
1
0
-1
N° Spikes/Cell/min
NS
Control
Letm1 siRNA
C
2μM CCCP
50mV
20 sec

S.3

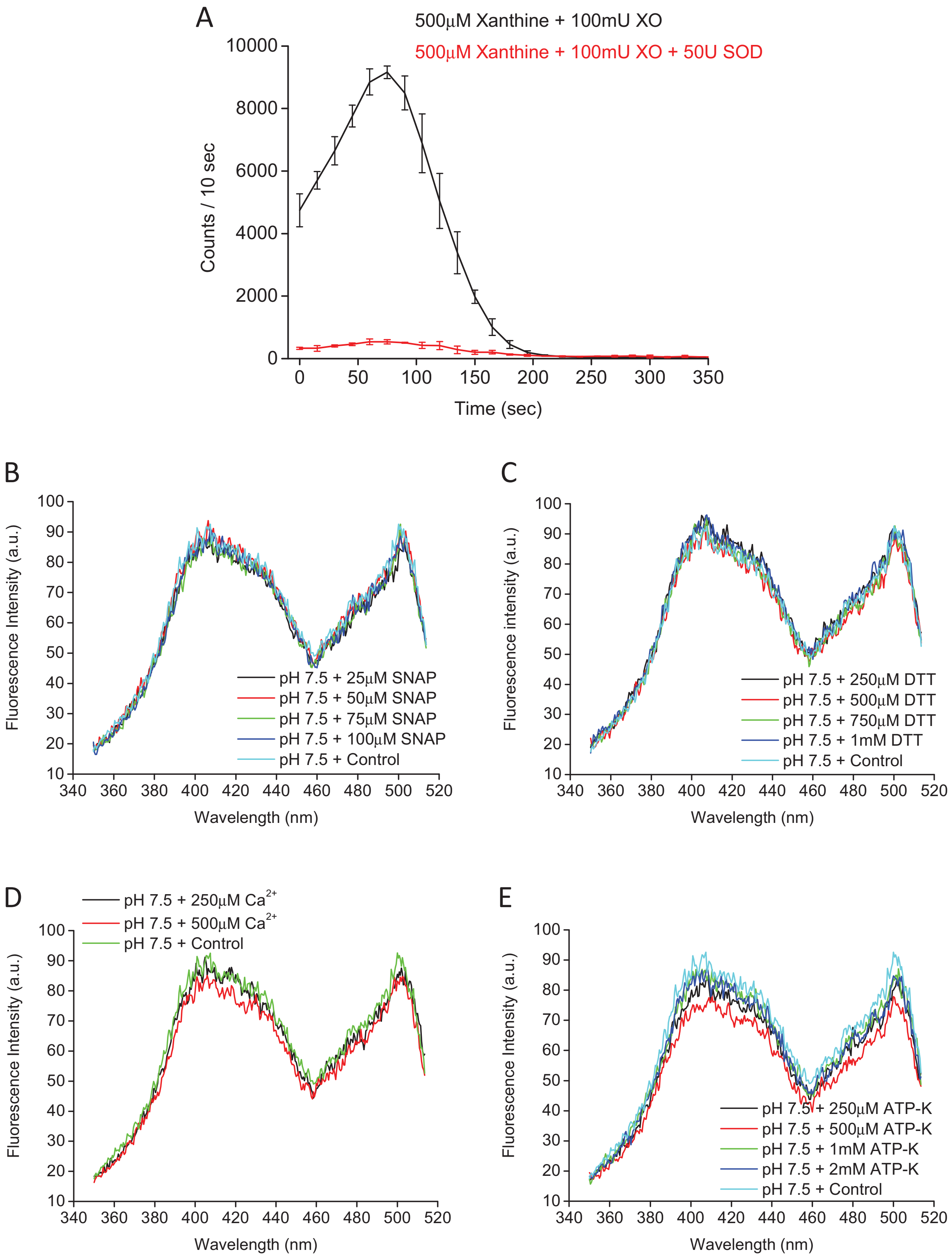
A
500μM Xanthine + 100mU XO
500μM Xanthine + 100mU XO + 50U SOD
Counts / 10 sec
Time (sec)
B
pH 7.5 + 25μM SNAP
pH 7.5 + 50μM SNAP
pH 7.5 + 75μM SNAP
pH 7.5 + 100μM SNAP
pH 7.5 + Control
Fluorescence Intensity (a.u.)
Wavelength (nm)
C
pH 7.5 + 250μM DTT
pH 7.5 + 500μM DTT
pH 7.5 + 750μM DTT
pH 7.5 + 1mM DTT
pH 7.5 + Control
Fluorescence intensity (a.u.)
Wavelength (nm)
D
pH 7.5 + 250μM Ca2+
pH 7.5 + 500μM Ca2+
pH 7.5 + Control
Fluorescence Intensity (a.u.)
Wavelength (nm)
E
pH 7.5 + 250μM ATP-K
pH 7.5 + 500μM ATP-K
pH 7.5 + 1mM ATP-K
pH 7.5 + 2mM ATP-K
pH 7.5 + Control
Fluorescence Intensity (a.u.)
Wavelength (nm)

S.4

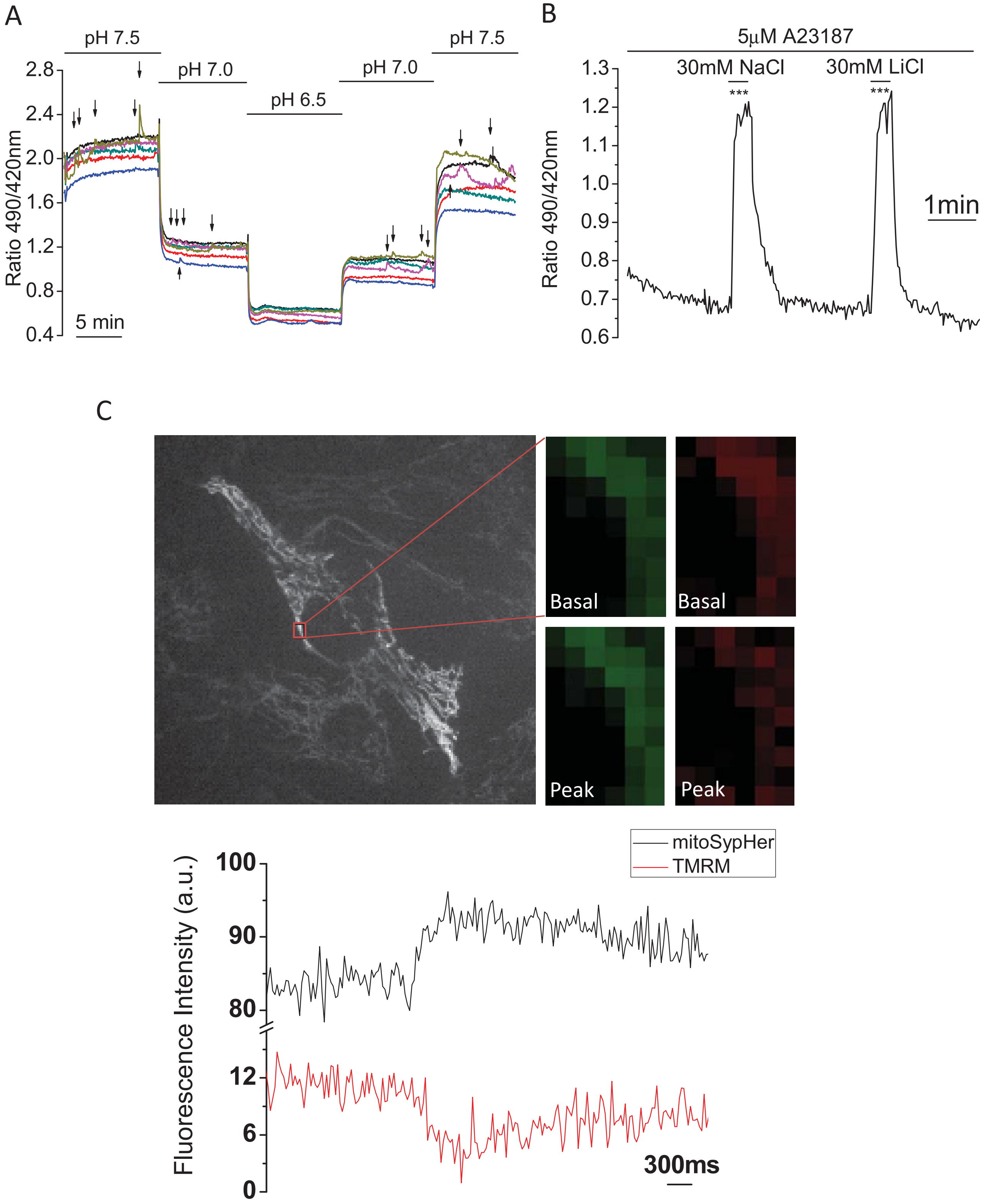
A
pH 7.5
pH 7.0
pH 6.5
pH 7.0
pH 7.5
Ratio 490/420nm
2.8
2.4
2.0
1.6
1.2
0.8
0.4
5 min
B
5µM A23187
30mM NaCl
30mM LiCl
***
***
Ratio 490/420nm
1.3
1.2
1.1
1.0
0.9
0.8
0.7
0.6
1min
C
Basal
Basal
Peak
Peak
mitoSypHer
TMRM
Fluorescence Intensity (a.u.)
100
90
80
12
6
0
300ms

S.5

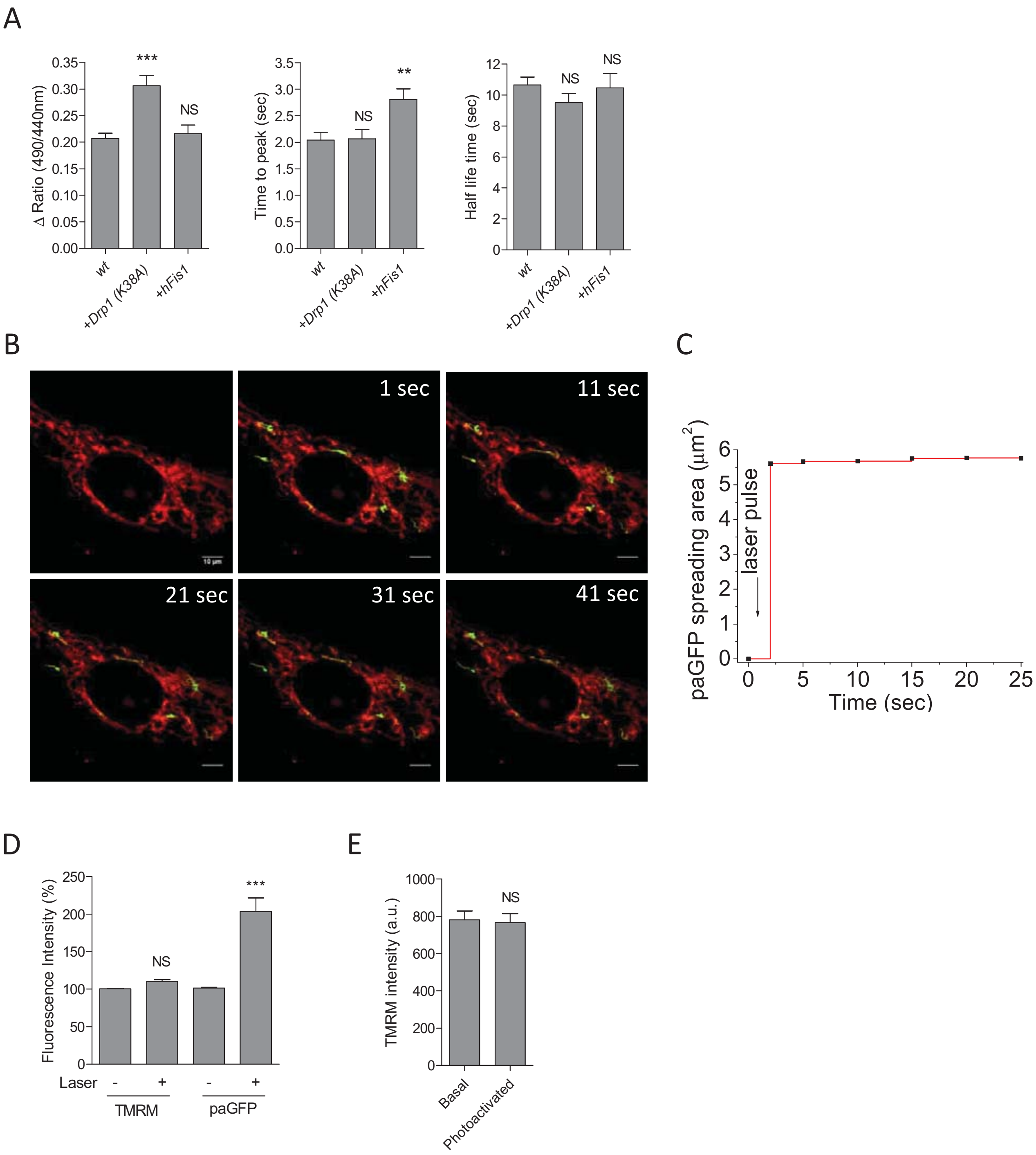

A
Δ Ratio (490/440nm)
0.35
0.30
0.25
0.20
0.15
0.10
0.05
0.00
***
NS
wt
+Drp1 (K38A)
+hFis1
Time to peak (sec)
3.5
3.0
2.5
2.0
1.5
1.0
0.5
0.0
NS
**
Half life time (sec)
12
10
8
6
4
2
0
NS
NS
B
1 sec
11 sec
21 sec
31 sec
41 sec
C
paGFP spreading area (μm²)
laser pulse
Time (sec)
0
5
10
15
20
25
D
Fluorescence Intensity (%)
250
200
150
100
50
0
NS
***
Laser
-
+
TMRM
paGFP
E
TMRM intensity (a.u.)
1000
800
600
400
200
0
NS
Basal
Photoactivated

S.6

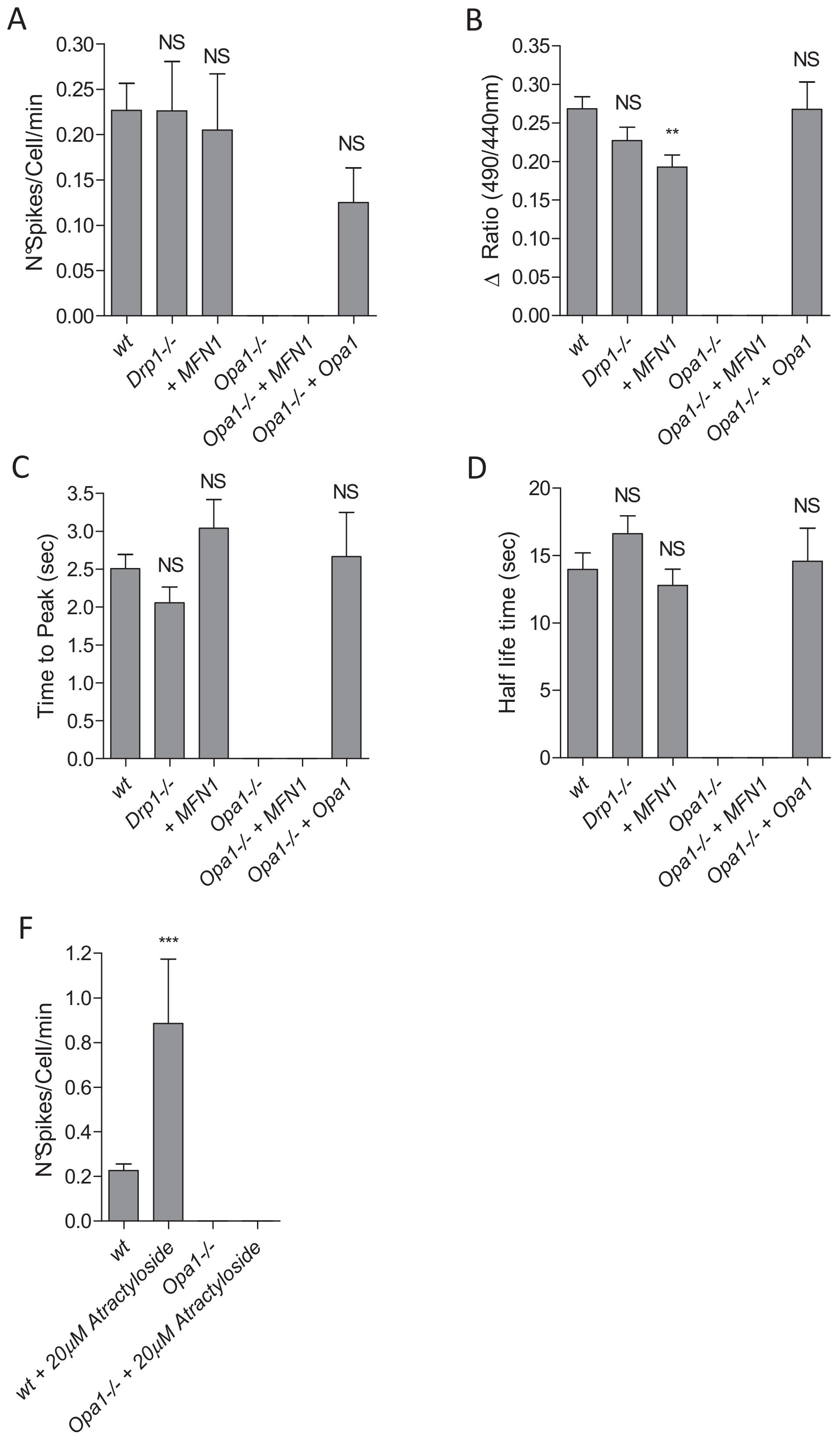

A
NS
NS
NS
N°Spikes/Cell/min
0.30
0.25
0.20
0.15
0.10
0.05
0.00
wt
Drp1-/-
+ MFN1
Opa1-/-
Opa1-/- + MFN1
Opa1-/- + Opa1
B
NS
**
NS
Δ Ratio (490/440nm)
0.35
0.30
0.25
0.20
0.15
0.10
0.05
0.00
wt
Drp1-/-
+ MFN1
Opa1-/-
Opa1-/- + MFN1
Opa1-/- + Opa1
C
NS
NS
NS
Time to Peak (sec)
3.5
3.0
2.5
2.0
1.5
1.0
0.5
0.0
wt
Drp1-/-
+ MFN1
Opa1-/-
Opa1-/- + MFN1
Opa1-/- + Opa1
D
NS
NS
NS
Half life time (sec)
20
15
10
5
0
wt
Drp1-/-
+ MFN1
Opa1-/-
Opa1-/- + MFN1
Opa1-/- + Opa1
F
***
N°Spikes/Cell/min
1.2
1.0
0.8
0.6
0.4
0.2
0.0
wt
wt + 20μM Atractyloside
Opa1-/-
Opa1-/- + 20μM Atractyloside

S.7

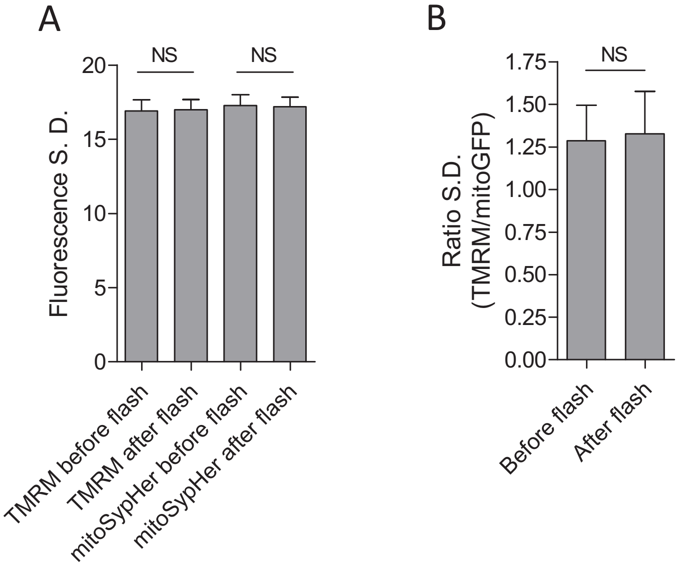
A
NS
NS
Fluorescence S. D.
20
15
10
5
0
TMRM before flash
TMRM after flash
mitoSypHer before flash
mitoSypHer after flash
B
NS
Ratio S.D.
(TMRM/mitoGFP)
1.75
1.50
1.25
1.00
0.75
0.50
0.25
0.00
Before flash
After flash
