# Supplementary material for: OPA1 promotes pH flashes that spread between contiguous mitochondria without matrix protein exchange
Source: EMBO J. 2013 May 28;32(13):1927–40. doi: 10.1038/emboj.2013.124 (PMC3981180; doi:10.1038/emboj.2013.124)
Supplement: Review Process File [file emboj2013124s9.pdf]

THE
EMBO
JOURNAL

Manuscript EMBO-2012-83602

OPA1 promotes pH flashes that spread between contiguous mitochondria without matrix protein exchange

Jaime Santo-Domingo, Marta Giacomello, Damon Poburko, Luca Scorrano, and Nicolas Demaurex

Corresponding author: Nicolas Demaurex, University of Geneva

Review timeline:

Submission date:	12 October 2012
Editorial Decision:	19 November 2012
Revision received:	27 February 2013
Additional Editorial Correspondence :	09 April 2013
Additional Author Correspondence :	15 April 2013
Accepted:	19 April 2013

Transaction Report:

(Note: With the exception of the correction of typographical or spelling errors that could be a source of ambiguity, letters and reports are not edited. The original formatting of letters and referee reports may not be reflected in this compilation.)

Editor: Anne Nielsen

1st Editorial Decision 19 November 2012

Thank you for submitting your manuscript for consideration by The EMBO Journal. It has now been seen by three referees whose comments are shown below.

As you will see from the reports, while all three referees express interest in your findings, they also raise a number of experimental concerns that you will need to address in full before submitting a revised version of the manuscript. We would especially emphasize that both referees #1 and #2 question the image quality and conclusiveness of the experimental data presented in figures 5 and 6 and that referee #1 asks you to directly measure mitochondrial membrane potential prior to and following fusion in order to support the proposed model. In addition, although we understand that it may be outside the scope of your work to provide a common explanation for all previous reports on mitochondrial flashes, we would ask you to address the issue further as suggested by referee #1.

Given the referees' positive recommendations, we offer you the opportunity to submit a revised version of the manuscript, addressing the comments of all three reviewers. I should add that it is EMBO Journal policy to allow only a single round of revision, and acceptance or rejection of your manuscript will therefore depend on the completeness of your responses to the full satisfaction of all three referees in this revised version. Please do not hesitate to contact me if you have questions related to the review process and the requests made by the referees.

When preparing your letter of response to the referees' comments, please bear in mind that this will form part of the Peer-Review Process File, and will therefore be available online to the community. For more details on our Transparent Editorial Process, please visit our website: http://www.nature.com/emboj/about/process.html

We generally allow three months as standard revision time. As a matter of policy, competing manuscripts published during this period will not negatively impact on our assessment of the conceptual advance presented by your study. However, we request that you contact the editor as soon as possible upon publication of any related work, to discuss how to proceed.

Thank you for the opportunity to consider your work for publication. I look forward to your revision.

REFEREE REPORTS:

Referee #1:

This is an intriguing paper which deals with the nature of transient depolarisations and pH changes in mitochondria. It is proposed that these reflect fusions of contiguous but discontinuous mitochondrial compartments mediated by Opa1 and that the pH change reflects a respiratory compensatory mechanism to adjust or equilibrate these different compartments. The paper is nicely (but not always clearly) presented and makes a potentially interesting story.

The observation of mitochondrial transient depolarisations, flashes or flickers has been kicking around for a long time. It has been odd that almost every group that has studied the events has arrived at a different mechanism. I did hope when I saw the title of this paper that this might provide a unifying mechanism, but more on that below.

The key observations here seem to be:
i) transient depolarisations are matched exactly by transient alkalinisations. This is not entirely new, especially if we consider that the data claiming to show superoxide flashes probably show transient alkalinisations (see Schwartzlander as cited);
ii) a new probe is described for mitochondrial matrix pH and shown to be insensitive to superoxide;
iii) The mitochondrial structures that disappear during transient depolarisations are not identical to structures filled with paGFP, suggesting the presence of contiguous but discontinuous matrix compartments within the apparent mitochondrial structures.
iv) the transients disappear when Opa 1 is knocked down.

I am left with several substantial questions and concerns about this interpretation:
The logic is that mitochondria of different potentials undergo fusion without the exchange of matrix contents but with some kind of electrical junction or fusion pore that allows equilibration of potentials sufficient to stimulate respiration and so drive the change in matrix pH.
i) If this were correct, perhaps it should it be possible to see potential differences between adjacent mitochondria associated with the flashes? Indeed - a requirement is that there must be substantial heterogeneity between mitochondrial membrane potentials within the population. It should be straightforward to demonstrate this. There appears to be significant heterogeneity of pH judging from the movies.
ii) In the final model, the structure with a high potential will depolarise but the structure with the lower potential will hyperpolarise. Is this ever seen? You would surely expect that for every depolarising event a nearby hyperpolarising event should be seen? Similarly, upon fusion, the mitochondrion with a higher potential will depolarise, stimulating respiration and driving the alkalinisation, but the fusing mitochondrion with a lower potential will increase its mean potential and so decrease its respiratory rate with an associated acidification. This also surely ought to be measurable?
iii) Why would the pH or potential flicker and show repetitive transients at the same spot? Surely you might reasonably expect from one fusion event a slower re-equilibration of potential and associated alkalinisation? If you argued that the putative channel or fusion pore openings flicker (as many channels do..) then the pH and potential transient changes at a single site should

systematically get progressively smaller as the potential difference gradually equilibrates. This doesn't seem to happen.
iv) Why would these happen repeatedly at the same location?
v) If the events serve to maintain bioenergetic competence, then knock out of OPA1 should be associated with an increase in the heterogeneity of potentials through the mitochondrial population. This should be measurable and would help confirm the story.
vi) The idea that the flashes represent fusion between compartments is based on the dissociation between the paGFP distribution and the distribution of potential changes - isn't it possible that the paGFP as a very large molecule may just diffuse much more slowly through the matrix dcompared to the almost instaneous changes in potential and/or pH? This is also not very well explained or illustrated and needs some work.
vii) OPA1 ko prevents pH flashes. What about mitochondrial potential flickers?

It seems bizarre that the potential flickers have been ascribed to so many different processes in many different cell types. It would be nice to find a unifying mechanism that explains these discrepancies as each publication has individually been quite convincing. Is there a role for calcium in OPA1 actions? Or oxidative stress? Is there some way that these disparate observations can be reconciled through one common process?

There are rather a lot of errors in this Ms for comfort. Most notably perhaps fig 4E doesn't exist. Mfn1 is spelled Mnf1.

General comments: the introduction is very long and very basic - I'm not sure this background is strictly necessary these days.

1. The material related to the paGFP (Figs 5/6) is really not explained very well at all. In the text, the authors talk about the paGFP illuminated area. It becomes clear if one looks at the movie etc that the photoactivated area is very small but this is very poorly explained and poorly illustrated. It is partly a matter of language - the word illuminated is used to explain changes in the GFP signal. How big was the area illuminated by the photoactivating light and how big the responsive element seem two different things. This is not at all clear. Even looking at the images, it is hard to see in the paGFP images exactly what has changed. I would strongly recommend some image processing to help illustrate the point - perhaps use a running differential to show pixels in which the signals have changed, or ratio the images against a basal image etc to show proportional changes in signal.

2. Fig 5C: This experiment is hard to read (as explained above), but also seems to have been done once... This seems very anecdotal and not a strong case. Further, the explanations are very muddled, as the authors write that they intended to study changes in 'paGFP during spontaneous decreases in m' and yet a few lines further down state that the loss of potential was 'possibly triggered by the laser illumination'... is it possible that all the signals are triggered by illumination and that this represents phototoxicity? And that the role of Opa1 is mediated by redox modulation as recently proposed by one of the authors?

3. I am worried by a few aspects of the Methods. 'For simultaneous pHmito/Ca2+ mito measurements, cells were incubated at room temperature for 30min with 2 mM Rhod-2-AM, washed for 20min, and imaged immediately'. This is a very high concentration of rhod-2AM - is this an error?

4. 'For pHmito/ m recordings, cells were incubated at room temperature for 5 min with 2 M Tetramethylrhodamine, methyl ester (TMRM), washed, and kept at 37{degree sign}C on the microscope until
signal reached stability.' Again, this is a rather bizarre protocol, and is a condition in which the TMRM at this very high concentration tends to induce phototoxicity. TMRM is more usually used at nM concentrations. Why was this protocol used?

5. I am also worried about the conditions used for permeabilised experiments:
'Cells were permeabilized by a short exposure to digitonin (1 min, 100 M) in a buffer containing 120 mM KCl, 10 mM NaCl, 1 mM H2KPO4, 20 mM HEPES, 5 mM succinic acid, 1 mM ATP-Mg2+, 0.02 mM ADP-K, 1 mM MgCl2, 0.5 mM EGTA adjusted to pH 7.4 with KOH. The ion free solution contained 10 mM HEPES, 5 mM succinic acid, 0.5 mM EGTA, and sucrose to reach 300

mOsm at pH 7.4.'
It is usual when using succinate as substrate to add rotenone to prevent reverse electron flow from complex II to I, generating ROS. I wonder why this was not done? Under the conditions described, it seems quite possible that the mitochondrial potential will have been maintained by the ATPase given this very high ATP concentration, in which case it may not be surprising that no flashes were seen.

Referee #2:

The paper is interesting and identifies an Opa1-dependent mechanism of mitochondrial changes in pH and subsequent membrane potential that is passed along semi-fused mitochondria. The use of the mito-SypHer is well controlled and proven that it can reliably measure pH and not be affected by ROS, Ca2+, etc.

A couple of comments:

#1. The statement in the introduction is inaccurate: "The sum of m and pHm generates a proton-motive force of -220 mV that powers ATP synthesis and that drives the transport of ions" The value of -220mV is reached typically under state 4 conditions (no ATP synthesis, low respiration rates). Therefore, -220mV pmf is not powering ATP synthesis, this -220mV is the value associated to basal proton conductance, respiration independent of ATP synthesis and thus represents an adaptation to lack of ATP synthesis and decreased proton re-entry.

#2. It is not clear why current knowledge in fusion and fission
challenges the maintenance of this permeability barrier to protons in the mitochondria of intact cells, as stated in the introduction. A pore generated between two adjacent inner membranes can occur without altering the permeability of the rest of the inner membrane, as the fusion site is small compared to the rest of the organelle (as suggested in the final figure of the manuscript). Other membrane fusion processes are, by concept, much more challenging in terms of permeability. In the case of mitochondria, given that the inner membrane is organized as cristae and that the fusion site is a small area compared to the rest. Here the real bioenergetic challenge is the fusion of two mitochondria that have different values of membrane potential. What would be the bioenergetic properties of the
mitochondria (and deltaPH) resulting from fusion or fission?

#3. For the photactivation experiments in Figure 5 and 6, the authors should provide readouts of TMRM fluorescence before and immediately after photoactivations , in order to demonstrate that the laser photoactivation is not at toxic levels to the mitochondria.

#4. In Figure 5C, it is very hard to distinguish the photoactivated mitochondrial area in the paGFP images. By eye, it would be extremely hard to tell exactly how far the paGFP has diffused among the mitochondrial network. Can the authors provide better images or explain exactly how they used these images to distinguish the boundaries of the photoactivated areas?

#5. The authors should provide calculations of the proton motif force in the mitochondria in which the pH flashes were detected, given the association with changes in membrane potential (under basal and under treatment with FCCP and Oligomycin). This, together with treatments of different inhibitors, would help to predict the respiratory state at which the mitochondria showing pH flashes are. In addition, respirometry should be performed to confirm that the FCCP dose used is increasing respiration. FCCP at a very high dose (not increasing respiration) can affect the acidification of many different compartments.

#6. The authors should discuss why Oligomycin decreases the pH in the matrix and the flashes, whereas atractyloside increases flashes. Both conditions are associated with increased membrane potential (and maybe alkalinazation of the matrix) and decreased respiration by affecting ATP synthesis. Why do they show such a difference in pH flashes?

#7. Long term Opa1 and Drp1 inactivation (24-48 hours) have strong effects on mitochondrial bioenergetics/function. Therefore, the differences seen in pH changes or protein content diffusion

might not be related to morphology, but to accumulation of mitochondria with affected respiratory chain function. Is there a more acute way to alter mitochondrial fusion/fission?

Referee #3:

The manuscript by Santo-Domingo and colleagues reports findings that changes in pH (flashes) are spread between mitochondria in a process that requires the inner membrane fusion mediator, Opa1. The authors use a pH sensitive fluorescent protein to clearly show this phenomenon. Moreover the authors show that the flashes propagate without mixing of matrix contents. Importantly, the authors show that the flashes observed are due to pH changes rather than superoxide production. The work therefore indicates that spreading of pH flashes is most likely achieved through the formation of a transient "fusion pore" formed between adjacent mitochondria that does not necessarily require complete fusion and mitochondrial mixing. The work is novel and highly interesting to a broad readership as it provides important new insights into mitochondrial communication and the relay of bioenergetic signals to the mitochondrial population. The work also provides new understanding into the process of transient fusion events. I have a few concerns that the authors should address.

Specific comments:
1.Details regarding the statistical analysis and what error bars in the figures indicate are missing.
2.Some of the micrographs with fluorescent images have had the original scale bar stamp covered by a black box and a new bar and label added over the top. I would favor that the originals are kept.
3.In Fig. 6A and B, I assume that the scale bars are supposed to represent 10 m, however I believe that some may be incorrect since the size of the nuclei differ between cells (e.g. Opa1-/- + OPA1).
4.The resolution of some of the images in Fig. 6A and 6B is quite poor. To help the readership, the authors should present clearer images of mitochondrial connectivity for Opa1-/- + OPA1. Mitochondrial lengths for this cell lines should also be shown in Fig. 6E.
5.The legend in Figure 6 should read Mfn1 not Mnf1.
6.It is interesting that atractyloside increased the pH flash frequency by about 5-fold (fig. 2I) and the authors do not have a clear answer as to why. Since the authors conclude that activity depends on Opa1's presence and membrane fusion, the authors could strengthen their conclusion by demonstrating that atractyloside does not cause pH flashes in Opa1-/- cells.

1st Revision - authors' response — 27 February 2013

The comments of the reviewers are in *italics*, our answers to these comments in plain letters

Referee #1:

I am left with several substantial questions and concerns about this interpretation: The logic is that mitochondria of different potentials undergo fusion without the exchange of matrix contents but with some kind of electrical junction or fusion pore that allows equilibration of potentials sufficient to stimulate respiration and so drive the change in matrix pH.
i) If this were correct, perhaps it should it be possible to see potential differences between adjacent mitochondria associated with the flashes? Indeed - a requirement is that there must be substantial heterogeneity between mitochondrial membrane potentials within the population. It should be straightforward to demonstrate this. There appears to be significant heterogeneity of pH judging from the movies.

We have measured the distribution of $\Delta\Psi_m$ within mitochondrial populations as well as the $\Delta\Psi_m$ changes occurring in adjacent mitochondria during flashes. The new Figure 7 shows that *Opa1* ablation markedly increased the heterogeneity of ΔΨm within the mitochondrial population of individual cells (panels A-C), and that the membrane potentials of adjacent mitochondria equilibrates after a flash (panels D-G), as discussed in more detail below.

ii) In the final model, the structure with a high potential will depolarize but the structure with the lower potential will hyperpolarize. Is this ever seen? You would surely expect that for every depolarizing event a nearby hyperpolarizing event should be seen? Similarly, upon fusion, the mitochondrion with a higher potential will depolarize, stimulating respiration and driving the

alkalinisation, but the fusing mitochondrion with a lower potential will increase its mean potential and so decrease its respiratory rate with an associated acidification. This also surely ought to be measurable?

Indeed, we observed that~66% of depolarizing events were associated with hyperpolarization events. We had missed these hyperpolarization events initially because they did not occur within the flashing mitochondria as we inferred but in adjacent mitochondria and we had to perform ratiometric imaging of TMRM over matrix-targeted GFP (or SypHer) to resolve them. An example of synchronous, opposite changes in membrane potential occurring in contiguous mitochondria is shown in Figure 7D. The hyperpolarization events were not associated with changes in pH_{mito} (Fig. 7E) and occurred in ~50% of adjacent mitochondria (Fig. 7F). They were never observed in mitochondria that were not in direct contact with a flashing unit, indicating that they did not reflect passive TMRM dye transfer. Importantly, the membrane potentials of the adjacent mitochondria equilibrated after the event, as predicted by our model (Fig. 7D and 7G). On average, the potential difference between adjacent mitochondria undergoing opposite changes in $\Delta\Psi_m$ decreased by ~40% after the flash (Fig. 7G). These data strengthen our model by showing that opposite changes in membrane potential occur between adjacent mitochondria and promotes their energy equilibration. We had to refine our model to account for the lack of pH flash propagation in hyperpolarizing mitochondria, and propose that this is due to the inhibition of proton pumping in the mitochondria with a lower potential.

iii) Why would the pH or potential flicker and show repetitive transients at the same spot? Surely you might reasonably expect from one fusion event a slower re-equilibration of potential and associated alkalinisation? If you argued that the putative channel or fusion pore openings flicker (as many channels do..) then the pH and potential transient changes at a single site should systematically get progressively smaller as the potential difference gradually equilibrates. This doesn't seem to happen.

We do indeed observe dissipating consecutive flashes, as illustrated in the two recordings below, but flashes of identical magnitude occurring repeatedly within the same mitochondrial structure were more frequent. One possible explanation for the lack of progressive equilibration is that the transient opening of a fusion pore might not allow long-term potential equilibration between mitochondria of different respiratory states. Indeed, our model predicts that the transiently coupled mitochondria would tend to regain their individual energetic status as soon as the pore closes. Although we could document that the membrane potential of adjacent mitochondria equilibrated immediately after the flash (Fig. 7D and 7G) this effect might be quite transient and we do not have evidence for or against long-term potential equilibration, apart from the global effect of *Opa1* ablation on the heterogeneity of membrane potentials (Fig. 7 A-C). It could be that the opening of a fusion pore fails to re-energize some adjacent mitochondria, the repetitive depolarization events thus reflecting failed attempt to restore energy status in damaged mitochondria. Further experiments are required to clarify this point, but we believe that these are outside the scope of this study.

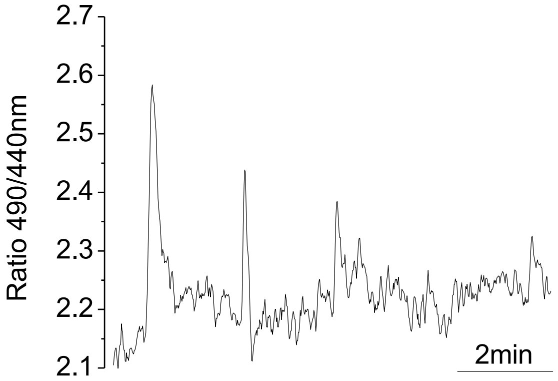

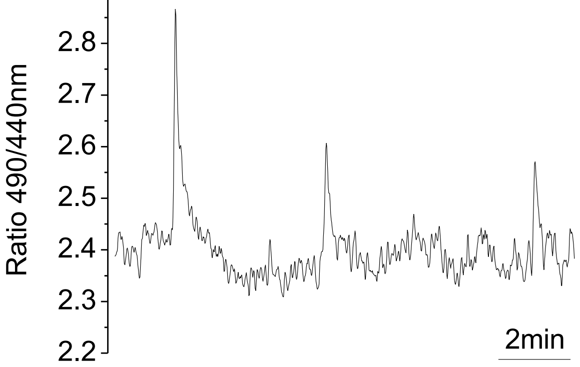

iv) Why would these happen repeatedly at the same location?

The flashes did not necessarily occur at the same location within cells as flashing mitochondria also moved, but we frequently observed repetitive events occurring within the same mitochondrial unit. We believe that these repetitive flashes reflect the flickering activity of a fusion pore forming at sites of OPA1-mediated inner membrane fusion. Why the mobility of these pore-forming sites is restricted is an interesting question. It could be that mitochondria attempting to fuse their inner membranes are locked together by the prior fusion of their outer membrane. We now mention that OPA1-mediated- flickering differs from the "kiss-and-run" mode of transient mitochondrial fusion previously reported (Liu et al, 2009), which allows exchange of soluble matrix proteins and promotes mitochondrial mobility (p.15).

v) If the events serve to maintain bioenergetic competence, then knock out of OPA1 should be associated with an increase in the heterogeneity of potentials through the mitochondrial population. This should be measurable and would help confirm the story.

The new Figure 7 (Panels A-C) shows that the heterogeneity of potentials through the mitochondrial population is markedly increased by *Opa1* ablation. We thank the reviewer for suggesting this experiment, which clearly shows that OPA1 promotes potential equilibration.

vi) The idea that the flashes represent fusion between compartments is based on the dissociation between the paGFP distribution and the distribution of potential changes - isn't it possible that the paGFP as a very large molecule may just diffuse much more slowly through the matrix compared to the almost instantaneous changes in potential and/or pH? This is also not very well explained or illustrated and needs some work.

We have verified that the rates of paGFP diffusion were not limiting the size of the compartment revealed by the photo conversion. Figs S5B and S5C show that paGFP spread within two seconds to cover a maximal area within interconnected mitochondria. This indicates that the matrix diffusion of paGFP is fast and is not limiting our ability to map the size of individual matrix compartments.

vii) OPA1 ko prevents pH flashes. What about mitochondrial potential flickers?

We now mention that *Opa1* ablation also abrogates the mitochondrial potential flickers (p. 11)
It seems bizarre that the potential flickers have been ascribed to so many different processes in many different cell types. It would be nice to find a unifying mechanism that explains these discrepancies as each publication has individually been quite convincing. Is there a role for calcium in OPA1 actions? Or oxidative stress? Is there some way that these disparate observations can be reconciled through one common process?

We now discuss how our results can reconcile previous reports of mitochondrial potential flickers (p.15). One key point is that our model implies the opening of a conductance between two mitochondria, connecting their matrix compartments. Previous models have implicated the opening of ion channels, transporters, or large conductance pores between the matrix and the IMS/cytosol. Our model therefore explains why flash activity is not coupled to ion fluxes and why mitochondria can preserve their bioenergetics competence during the flashes, as connecting two matrixes will not dissipate the proton-motive force. By showing that the flash activity requires OPA1-mediated fusion (which we now further illustrate by showing the temporal correlation between mitochondrial contact and flash activity, Fig. 5C), we link flash activity to a highly regulated cellular process. At present, there is no evidence that calcium directly regulates the fusion process, as mitochondrial fusion and PARL rhomboid activity *in vitro* do not require Ca^{2+} (for review, see (Jeyaraju et al, 2009), but Ca^{2+} controls DRP1 recruitment (Cereghetti et al, 2008) and MFN1ubiquitination (Ziviani et al, 2010), thus indirectly regulating the fusion process. There are numerous reports that ROS levels control mitochondrial dynamics, and *Opa1* mutations have been linked to elevated ROS production and reduced lifespan in Drosophila (Tang et al, 2009). These mechanisms could explain earlier reports that calcium and ROS drive flashing activity. We will be happy to discuss how all prior findings can be integrated in the framework of a unifying model, but believe that this would require an extensive and rather theoretical discussion that would be better suited for a review article.

© EMBO

There are rather a lot of errors in this Ms for comfort. Most notably perhaps fig 4E doesn't exist. Mfn1 is spelled Mnf1.

We apologize for these errors that have been corrected. We now abide to the standard nomenclature, i.e. UPPERCASE for proteins and *lowercase italics* for genes.

General comments: the introduction is very long and very basic - I'm not sure this background is strictly necessary these days.

The introduction has been shortened by 15%

1. The material related to the paGFP (Figs 5/6) is really not explained very well at all. In the text, the authors talk about the paGFP illuminated area. It becomes clear if one looks at the movie etc. that the photoactivated area is very small but this is very poorly explained and poorly illustrated. It is partly a matter of language - the word illuminated is used to explain changes in the GFP signal. How big was the area illuminated by the photoactivating light and how big the responsive element seem two different things. This is not at all clear. Even looking at the images, it is hard to see in the paGFP images exactly what has changed. I would strongly recommend some image processing to help illustrate the point - perhaps use a running differential to show pixels in which the signals have changed, or ratio the images against a basal image etc. to show proportional changes in signal.

We have improved the presentation and the discussion of these figures. The previous images were in fact F/F0 as suggested above but this mode of proportional presentation poorly illustrates the size of the compartments containing the photoconverted GFP. Our intention was not to illustrate the speed of paGFP diffusion, which as discussed above is too fast to be resolved (Fig S5), but to map the size of the individual matrix compartments labeled with the photoactivated protein. We have replaced all the paGFP images with merged TMRM/paGFP images, to better distinguish the mitochondrial network (in red) from the individual matrix compartments labeled with paGFP (in green). We also show insets to show the regions containing the paGFP at higher magnification and hope that this mode of presentation allows a better comparison of mitochondrial connectivity in the different conditions. We have also revised the text to avoid ambiguous terms such as "illuminated" or "photoactivated" and refer to regions containing the photoactivated protein as "paGFP-labeled" areas.

2. Fig 5C: This experiment is hard to read (as explained above), but also seems to have been done once... This seems very anecdotal and not a strong case. Further, the explanations are very muddled, as the authors write that they intended to study changes in 'paGFP during spontaneous decreases in ΔΨm' and yet a few lines further down state that the loss of potential was 'possibly triggered by the laser illumination'... is it possible that all the signals are triggered by illumination and that this represents phototoxicity? And that the role of Opa1 is mediated by redox modulation as recently proposed by one of the authors?

This experiment is technically challenging and we have only a handful of recordings because the regions chosen for photoactivation had to be defined before starting the experiment on our confocal microscope. Since the flashes occur randomly in time and space, we could only obtain data when repetitive flashes occurred within the regions selected for photoconversion. In the example shown the TMRM drop occurred immediately after the photoactivation, prompting the comment that this particular event was possibly triggered by the illumination. However, at least one TMRM drop occurred before laser irradiation in these recordings, and we have examples of photoconversion triggered after the occurrence of the TMRM drop. We are confident that the TMRM drops are not triggered by laser illumination, as they occurred spontaneously without laser illumination on conventional fluorescence microscopes. We have removed this figure intending to illustrate graphically that the paGFP compartment was smaller than the compartment undergoing pH_{mito} or $\Delta\Psi_m$ fluctuations, a point that we feel is better documented by the quantitative data of Fig. 5A and 5B. Instead, we include in Fig. 5C a figure that, together with a supplementary movie, illustrates the temporal correlation between mitochondrial fusion events and pH_{mito} flash activity.

3. I am worried by a few aspects of the Methods. 'For simultaneous pHmito/Ca2+ mito measurements, cells were incubated at room temperature for 30min with 2 mM Rhod-2-AM, washed

for 20min, and imaged immediately'. This is a very high concentration of rhod-2AM - is this an error?

The concentration used was 2 µM, not 2 mM. We apologize for this typo

4. 'For pHmito/Ψm recordings, cells were incubated at room temperature for 5 min with 2 µM Tetramethylrhodamine, methyl ester (TMRM), washed, and kept at 37{degree sign}C on the microscope until signal reached stability.' Again, this is a rather bizarre protocol, and is a condition in which the TMRM at this very high concentration tends to induce phototoxicity. TMRM is more usually used at nM concentrations. Why was this protocol used?

This protocol was used in initial experiments, but in all the experiments illustrated TMRM was used at a concentration of 4 nM, added 20 min before and present throughout the recording. We apologize for this confusion.

5. I am also worried about the conditions used for permeabilised experiments: 'Cells were permeabilized by a short exposure to digitonin (1 min, 100 µM) in a buffer containing 120 mM KCl, 10 mM NaCl, 1 mM H2KPO4, 20 mM HEPES, 5 mM succinic acid, 1 mM ATP-Mg2+, 0.02 mM ADP-K, 1 mM MgCl2, 0.5 mM EGTA adjusted to pH 7.4 with KOH. The ion free solution contained 10 mM HEPES, 5 mM succinic acid, 0.5 mM EGTA, and sucrose to reach 300 mOsm at pH 7.4.' It is usual when using succinate as substrate to add rotenone to prevent reverse electron flow from complex II to I, generating ROS. I wonder why this was not done? Under the conditions described, it seems quite possible that the mitochondrial potential will have been maintained by the ATPase given this very high ATP concentration, in which case it may not be surprising that no flashes were seen.

We did not add rotenone because it inhibits the flash activity (Fig 2). Reverse activity of the ATPase is unlikely as ATP was not included in the ion-free solution indicated above, yet robust activity was observed in this condition (Fig. 4A).

Referee #2:

The paper is interesting and identifies an Opa1-dependent mechanism of mitochondrial changes in pH and subsequent membrane potential that is passed along semi-fused mitochondria. The use of the mito-SypHer is well controlled and proven that it can reliably measure pH and not be affected by ROS, Ca2+, etc.

A couple of comments:

#1. The statement in the introduction is inaccurate: "The sum of ΔΨm and ΔpHm generates a proton-motive force of -220 mV that powers ATP synthesis and that drives the transport of ions" The value of -220mV is reached typically under state 4 conditions (no ATP synthesis, low respiration rates). Therefore, -220mV pmf is not powering ATP synthesis, this -220mV is the value associated to basal proton conductance, respiration independent of ATP synthesis and thus represents an adaptation to lack of ATP synthesis and decreased proton re-entry.

This sentence has been removed as the introduction has been condensed on reviewer #1 suggestion

#2. It is not clear why current knowledge in fusion and fission challenges the maintenance of this permeability barrier to protons in the mitochondria of xintact cells, as stated in the introduction. A pore generated between two adjacent inner membranes can occur without altering the permeability of the rest of the inner membrane, as the fusion site is small compared to the rest of the organelle (as suggested in the final figure of the manuscript). Other membrane fusion processes are, by concept, much more challenging in terms of permeability. In the case of mitochondria, given that the inner membrane is organized as cristae and that the fusion site is a small area compared to the rest. Here the real bioenergetic challenge is the fusion of two mitochondria that have different values of membrane potential. What would be the bioenergetic properties of the mitochondria (and deltaPH) resulting from fusion or fission?

We now discuss more precisely how some mechanisms proposed for mitochondrial fusion challenge the preservation of the inner membrane ionic permeability (p. 5). Indeed, the opening of a fusion pore between two adjacent inner membranes does not challenge mitochondrial permeability during fusion, and we now mention that the real bioenergetic challenge is the equilibration of two mitochondria of different membrane potentials. Defective fusion pore assembly however could connect the matrix with the IMS. *In vitro* experiments indicate that OPA1 induces lipid tubulation (Ban et al, 2010), and rupture of the growing IMM tubules could link the matrix to the IMS. If pore formation involves the juxtaposition of two hemi-channels as for gap junctions, opening of the hemi-channels on the growing IMM tubule would also connect the matrix to the IMS. Such a conductance would dissipate the proton-motive force and compromise the maintenance of the mitochondrial ionic permeability.

#3. For the photactivation experiments in Figure 5 and 6, the authors should provide readouts of TMRM fluorescence before and immediately after photoactivations , in order to demonstrate that the laser photoactivation is not at toxic levels to the mitochondria.

We now provide fluorescence readouts of both TMRM and paGFP before and immediately after photoactivation to show that laser illumination was not toxic to mitochondria (Figs S5D and S5E).

#4. In Figure 5C, it is very hard to distinguish the photoactivated mitochondrial area in the paGFP images. By eye, it would be extremely hard to tell exactly how far the paGFP has diffused among the mitochondrial network. Can the authors provide better images or explain exactly how they used these images to distinguish the boundaries of the photoactivated areas?

We have removed this figure illustrating graphically that paGFP compartments are smaller than compartments undergoing pH flashes as we feel that this point is better documented by the quantitative data of Fig. 5A and 5B. To better distinguish the boundaries of the photoactivated areas we now show merged TMRM/paGFP images with the paGFP-labeled regions shown at higher magnification in insets (Figs. 5B and 6B)

#5. The authors should provide calculations of the proton motif force in the mitochondria in which the pH flashes were detected, given the association with changes in membrane potential (under basal and under treatment with FCCP and Oligomycin). This, together with treatments of different inhibitors, would help to predict the respiratory state at which the mitochondria showing pH flashes are. In addition, respirometry should be performed to confirm that the FCCP dose used is increasing respiration. FCCP at a very high dose (not increasing respiration) can affect the acidification of many different compartments.

We have attempted to calibrate our TMRM recordings with CCCP and oligomycin in order to provide estimates of the proton-motive force in the flashing mitochondria (p. 14). However, the changes in TMRM fluorescence as a function of voltage are not linear and our estimates are therefore quite imprecise. We conservatively estimate that $\Delta\Psi_m$ was around -120 mV at rest and decreased to -50 mV during a flash. The only value that we could precisely measure is the matrix pH, which averaged 7.6 before a flash and increased by 0.4 pH unit during the flash. The other component of ΔpH_m, the IMS pH, was previously measured at 6.8 in HeLa cells (Porcelli et al, 2005) but we do not know whether the IMS acidifies or not during a flash. Assuming that it does, an acidification of 0.4 pH unit that would match the matrix alkalinization during a flash seems reasonable. We therefore estimate that ΔpH_m is around 1 pH units at rest and increases to 1.8 pH units during a flash, generating a driving force of -60 mV and -110 mV respectively. Based on these calculations, the resting proton-motive force of -180 mV decreases by only ~20 mV during a flash, but the relative contributions of its electrical and chemical components become inverted. As suggested, we have performed respirometry to show that CCCP indeed increases respiration at the dose used (Fig. S2C)

#6. The authors should discuss why Oligomycin decreases the pH in the matrix and the flashes, whereas atractyloside increases flashes. Both conditions are associated with increased membrane potential (and maybe alkalinazation of the matrix) and decreased respiration by affecting ATP synthesis. Why do they show such a difference in pH flashes?

We now discuss in more detail the effects of oligomycin and atractyloside on the matrix pH (p. 15). We previously showed that oligomycin evokes biphasic changes in matrix pH, with an initial alkalinization reflecting ATP synthase inhibition and a secondary acidification reflecting pH equilibration between the matrix and the cytosol (Poburko et al, 2011). We agree that the diverging effects of oligomycin and atractyloside on the pH flash activity are difficult to explain by changes in bioenergetics parameters. Instead, we believe that they reflect the different effects of these inhibitors on the fusion process, possibly related to changes in matrix ATP. Oligomycin inhibits the ATP synthase while atractyloside inhibits the ANT, causing opposite changes in matrix ATP levels that might differently modulate the formation of a fusion pore. Regardless of the underlying mechanism, the effect of atractyloside requires OPA1 and, following the suggestion of reviewer #3, we now show that atractyloside does not evoke flash activity in *Opa1*-ablated cells, linking its stimulatory effects to OPA1-mediated fusion.

#7. Long term Opa1 and Drp1 inactivation (24-48 hours) have strong effects on mitochondrial bioenergetics/function. Therefore, the differences seen in pH changes or protein content diffusion might not be related to morphology, but to accumulation of mitochondria with affected respiratory chain function. Is there a more acute way to alter mitochondrial fusion/fission?

We are currently generating floxed mice that will allow acute ablation of key mitochondrial shaping proteins, but unfortunately these animals are not yet available. To ensure that the differences in pH flash activity and in paGFP diffusion that we report here were not due to gross alterations in mitochondrial bioenergetics, we have measured the resting potential and matrix pH of the knockout cells used here. These parameters were comparable between the different cell lines, indicating that the main mitochondrial bioenergetics parameters are preserved during long-term invalidation of *Opa1* and *Drp1*. As suggested by reviewer #1, we found that the heterogeneity of the mitochondrial membrane potential was increased by *Opa1* ablation (Fig. 7A-C), a finding that strengthens our proposal that the OPA1-mediated pH flashes promote energy equilibration between adjacent mitochondria.

Referee #3:

The manuscript by Santo-Domingo and colleagues reports findings that changes in pH (flashes) are spread between mitochondria in a process that requires the inner membrane fusion mediator, Opa1. The authors use a pH sensitive fluorescent protein to clearly show this phenomenon. Moreover the authors show that the flashes propagate without mixing of matrix contents. Importantly, the authors show that the flashes observed are due to pH changes rather than superoxide production. The work therefore indicates that spreading of pH flashes is most likely achieved through the formation of a transient "fusion pore" formed between adjacent mitochondria that does not necessarily require complete fusion and mitochondrial mixing. The work is novel and highly interesting to a broad readership as it provides important new insights into mitochondrial communication and the relay of bioenergetic signals to the mitochondrial population. The work also provides new understanding into the process of transient fusion events. I have a few concerns that the authors should address.

Specific comments:
1.Details regarding the statistical analysis and what error bars in the figures indicate are missing.

We now provide details as to how the statistical analysis was made and specify the nature of the error bars in the figures (p. 21). We apologize for this omission.

2.Some of the micrographs with fluorescent images have had the original scale bar stamp covered by a black box and a new bar and label added over the top. I would favor that the originals are kept.

We have kept the original scale bar stamp in all the paGFP confocal images.

3.In Fig. 6A and B, I assume that the scale bars are supposed to represent 10 μm, however I believe that some may be incorrect since the size of the nuclei differ between cells (e.g. Opa1-/- + OPA1).

Correct, we apologize for the mismatch. We have resized the images in Figs. 5 and 6 so that all scale bars of 10µm match and include insets (with identical scale bars) to show the paGFP-labeled compartments at higher magnification.

4.The resolution of some of the images in Fig. 6A and 6B is quite poor. To help the readership, the authors should present clearer images of mitochondrial connectivity for Opa1-/- + OPA1. Mitochondrial lengths for this cell lines should also be shown in Fig. 6E.

We have replaced the F/F0 paGFP images in Fig 6B and 5B with merged TMRM/paGFP images, with insets to show the individual matrix compartments labeled with the photoactivated protein at higher magnification. We hope that this mode of presentation better illustrates the mitochondrial connectivity of the different genotypes. We also include the mitochondrial length data for the *Opa1*$^{-/-}$ + OPA1condition in Fig. 6E.

5.The legend in Figure 6 should read Mfn1 not Mnf1.

Corrected, thank you. We now use the standard nomenclature for all proteins and genes, i.e. UPPERCASE and *lowercase italics*, respectively.

6.It is interesting that atractyloside increased the pH flash frequency by about 5-fold (fig. 2I) and the authors do not have a clear answer as to why. Since the authors conclude that activity depends on Opa1's presence and membrane fusion, the authors could strengthen their conclusion by demonstrating that atractyloside does not cause pH flashes in Opa1-/- cells.

Fig. S6E shows that atractyloside does not evoke flash activity in *Opa1*$^{-/-}$ cells. We thank the reviewer for suggesting this experiment that strengthens the link between pH flash activity and OPA1-mediated membrane fusion.
References cited:

Ban T, Heymann JA, Song Z, Hinshaw JE, Chan DC (2010) OPA1 disease alleles causing dominant optic atrophy have defects in cardiolipin-stimulated GTP hydrolysis and membrane tubulation. *Human molecular genetics* **19:** 2113-2122

Cereghetti GM, Stangherlin A, Martins de Brito O, Chang CR, Blackstone C, Bernardi P, Scorrano L (2008) Dephosphorylation by calcineurin regulates translocation of Drp1 to mitochondria. *Proceedings of the National Academy of Sciences of the United States of America* **105:** 15803-15808

Jeyaraju DV, Cisbani G, Pellegrini L (2009) Calcium regulation of mitochondria motility and morphology. *Biochimica et biophysica acta* **1787:** 1363-1373

Liu X, Weaver D, Shirihai O, Hajnoczky G (2009) Mitochondrial 'kiss-and-run': interplay between mitochondrial motility and fusion-fission dynamics. *The EMBO journal* **28:** 3074-3089

Poburko D, Santo-Domingo J, Demaurex N (2011) Dynamic regulation of the mitochondrial proton gradient during cytosolic calcium elevations. *The Journal of biological chemistry* **286:** 11672-11684

Porcelli AM, Ghelli A, Zanna C, Pinton P, Rizzuto R, Rugolo M (2005) pH difference across the outer mitochondrial membrane measured with a green fluorescent protein mutant. *Biochemical and biophysical research communications* **326:** 799-804

Tang S, Le PK, Tse S, Wallace DC, Huang T (2009) Heterozygous mutation of Opa1 in Drosophila shortens lifespan mediated through increased reactive oxygen species production. *PloS one* **4:** e4492

Ziviani E, Tao RN, Whitworth AJ (2010) Drosophila parkin requires PINK1 for mitochondrial translocation and ubiquitinates mitofusin. *Proceedings of the National Academy of Sciences of the United States of America* **107:** 5018-5023

© EMBO

Additional Editorial Correspondence 09 April 2013

Thank you for submitting your revised manuscript to The EMBO Journal and my apologies for the unusually long review period in this case.

We have now finally heard back from one of the original referees whose comments are included below; however, as you will see this person remains unconvinced that mitochondrial fusion events underlie the observed pH flashes. S/he therefore requests that you either provide additional experimental evidence or substantially alter the conclusions drawn in the manuscript.

Given this rather negative assessment - and to ensure that we are making a fair and balanced decision in this case - I will now contact one of the other original referees for a second opinion on your revised manuscript (they were not initially available to re-review). In addition, I would also like to give you the chance to see the existing report and to comment on the criticisms raised as well as potential data that could be supplied to address them.

REFEREE REPORT:

Referee #1:

While the authors have addressed most of the minor concerns, some major issues have not resolved:

- The major concern still present after this round of revision is that there is no direct evidence that the pH flashes are caused by an hypothetical transient pore that resembles gap junctions during a brief mitochondrial fusion event, as proposed by the authors.
Furthermore, no experiments were performed to show and/or directly quantify inner as well as outer membrane fusion and its correlation to a pH flash, which are necessary to prove the author's hypothesis. This could had been done by PEG cell fusion assays of two cells expressing differentially labeled outer or inner membrane proteins and see how outer and/or inner membrane protein transfer or transient
colocalization correlates to a pH flash (as the resolution of confocal fluorescence microscopy is around 200 nm). Therefore, the molecular mechanism causing the pH flashes has not been successfully identified.

-Despite being feasible, experiments acutely modulating mitochondrial fusion and fission events were not performed. Examples of experimental models currently available and previously published that acutely modulate fusion and fission rates are listed here: Mdivi treatment- Drp1 inhibitor-, nocodazole treatment to prevent mitochondrial movement and thus mitochondrial fusion. Other medium term experiments would be inducible siRNA for Mfns, Opa1, Drp1 or Cre in Mfn1, Mfn2 and Drp1 LoxP cells. However, these experiments would require some additional set up, as some effects could be caused by an alteration in mitochondrial quality control mechanisms and not just by decreased fusion and or fission events. The experimental models currently used in the paper (KO cells) cannot be used to prove the author's hypothesis, as lack of pH flashes can be caused by mitochondrial dysfunction induced by the genetic modifications used, by altered mtDNA homeostasis,and due to the long term inhibition of inter-mitochondrial fusion and complementation. Therefore, the precise molecular mechanism behind the pH flashes remains elusive.
If authors do not perform these experiments, I would recommend publishing this manuscript only if author's remove the model proposing that pH flashes are caused by a pore opening. The data only shows that pH flashes are dependent on the electron transport chain function and that modulation of mitochondrial homeostasis by changing fusion and fission proteins can regulate the frequency of these flashes.

Additional Author Correspondence 15 April 2013

I have now consulted with my co-authors who all agree that the new experiments requested by the critical reviewer will not improve our study and will not provide the requested proof. We understand that the reviewer asks us to validate our model by providing direct evidence that mitochondria are coupled by gap junctions. However, the experiments that he/she proposes will not provide definitive

© EMBO

proof of gap junction coupling. In our opinion, such proof will require sophisticated electrophysiological recordings of pairs of mitochondria, using two electrodes. While we understand that a revised manuscript is examined more critically as it is closer to publication, we find it unfair to propose experiments that will substantially delay its publication without providing the requested proof. Rather than performing these new experiments, we prefer to comply with the reviewer's request to revise our conclusions, and to stress in our discussion that the existence of fusion pores electrically coupling mitochondria remains hypothetical.

Accepted 19 April 2013

Thank you for submitting a revised version of your manuscript to The EMBO Journal. It has now been seen by all three of the original referees whose comments are shown below.

As you will see, the referees generally find that most major criticisms have been sufficiently addressed in this revised manuscript, and although some concerns remain about the possible pore fusion underlying formation of pH flashes, the referees all agree to recommend the manuscript for publication pending textual changes to emphasize the speculative nature of the model. In addition, referee #1 asks you to elaborate on the description of figures 5 and 7.

Given these positive recommendations by the referees, I am pleased to inform you that your manuscript has now been accepted for publication in the EMBO Journal pending the revisions outlined above.

REFEREE REPORTS:

Referee #1:

The rebuttal letter is quite convincing and the authors have clearly taken on board concerns and criticisms of the first submission, and have new data, which fits predictions that arose during the first review. However, one of these points was that OPA1 mediated fusion events between two mitochondria of different potentials should cause the potential of one to increase and the other to decrease potential so that both become the same. As I understand it, the image sequence used in support of this model (Fig 7D and the supplementary video) don't show this at all - rather they seem to show two structures with rather similar potential that undergo a flash in which one suddenly increases a bit whilst the other almost disappears, to gradually reappear later. While it is true that they equilibrate at the end, why should the potential of one decrease so much? The hyperpolarising structure doesn't start off depolarised and then increase potential to equilibrate with its neighbour, but starts off the same and then increases a bit. I find it very hard to understand how this can happen according to the model.

I have to confess also that I still struggle to understand exactly what was done during the paGFP experiments - it isn't clear to me what areas of the image were flashed and the GFP activated. I dare say I am being dim witted, but I think it needs to be clearer given the time most people are willing to give to reading a paper these days.

One of the co-referees doesn't like the pore model, and it has to be said that you have no direct evidence that there is a fusion pore. It would seem reasonable to make it clear that this is speculative. However I would imagine that theoretically you would need some kind of pore opening (according to this model) in order to generate a rapid transient that then relaxes - gradual membrane fusion would presumably just cause gradual equilibration.

Referee #2 :

While the authors have addressed most of the minor concerns, some major issues have not resolved:

- The major concern still present after this round of revision is that there is no direct evidence that the pH flashes are caused by an hypothetical transient pore that resembles gap junctions during a brief mitochondrial fusion event, as proposed by the authors. Furthermore, no experiments were

performed to show and/or directly quantify inner as well as outer membrane fusion and its correlation to a pH flash, which are necessary to prove the author's hypothesis. This could had been done by PEG cell fusion assays of two cells expressing differentially labeled outer or inner membrane proteins and see how outer and/or inner membrane protein transfer or transient colocalization correlates to a pH flash (as the resolution of confocal fluorescence microscopy is around 200 nm). Therefore, the molecular mechanism causing the pH flashes has not been successfully identified.

-Despite being feasible, experiments acutely modulating mitochondrial fusion and fission events were not performed. Examples of experimental models currently available and previously published that acutely modulate fusion and fission rates are listed here: Mdivi treatment- Drp1 inhibitor-, nocodazole treatment to prevent mitochondrial movement and thus mitochondrial fusion. Other medium term experiments would be inducible siRNA for Mfns, Opa1, Drp1 or Cre in Mfn1, Mfn2 and Drp1 LoxP cells. However, these experiments would require some additional set up, as some effects could be caused by an alteration in mitochondrial quality control mechanisms and not just by decreased fusion and or fission events. The experimental models currently used in the paper (KO cells) cannot be used to prove the author's hypothesis, as lack of pH flashes can be caused by mitochondrial dysfunction induced by the genetic modifications used, by altered mtDNA homeostasis,and due to the long term inhibition of inter-mitochondrial fusion and complementation. Therefore, the precise molecular mechanism behind the pH flashes remains elusive.

If authors do not perform these experiments, I would recommend publishing this manuscript only if author's remove the model proposing that pH flashes are caused by a pore opening. The data only shows that pH flashes are dependent on the electron transport chain function and that modulation of mitochondrial homeostasis by changing fusion and fission proteins can regulate the frequency of these flashes.

Referee #3:

I believe that the authors have responded well to the criticisms raised. While not all of the answers fully address the reviewers' concerns, I think the authors have made considered efforts to improve the manuscript with additional experimental data and (importantly) significant changes within the text. Some of the experimental approaches requested by the reviewers, are in my opinion, out of the realms of expectation and would take many months and significant resources to complete - and still would not lead to definitive results. With no disrespect to The EMBO Journal (which is a high quality journal), I also think that should the authors obtain results using additional sophisticated experiments, they would have pitched the manuscript to a higher-tier journal. In essence, I believe that the manuscript should be accepted for publication.

I agree that there are inherent issues with using the Opa1 knockout cell line which is known to cause mitochondrial bioenergetic defects. However, the known properties of Opa1 in mitochondrial inner membrane fusion is a strong indicator that this is the protein most likely involved in spreading pH flashes. Importantly, the authors show that the pH flashes are recovered upon Opa1 re-expression. How atractyloside is involved in stimulating pH flashes is something that I expect will lead to others to investigate.

I favour that the authors keep the model since it is an important summary of the work. The authors clearly state that this is a "proposed mechanism". It will be up to the scientific community to determine whether this is in fact a "pore". Identification of components of other controversial mitochondrial pores (e.g. related to apoptosis, calcium flow and mitochondrial permeability transitions) has aided scientific debate and research studies and I imagine this relatively controversial topic will be similar. A compromise could be to not show a pore that looks like a protein channel but instead place a question mark in a circle/box.

Additional Author Correspondence 25 April 2013

Thank you for informing us that our manuscript has been accepted for publication in the EMBO Journal pending textual changes. As requested, we have now edited our MS to emphasize the speculative nature of the model, provide a more detailed description of figures 5 and 7, and specify the nature of the error bars and the number of replicas used for calculating statistics in all relevant figures.

Referee #1:

The rebuttal letter is quite convincing and the authors have clearly taken on board concerns and criticisms of the first submission, and have new data, which fits predictions that arose during the first review. However, one of these points was that OPA1 mediated fusion events between two mitochondria of different potentials should cause the potential of one to increase and the other to decrease potential so that both become the same. As I understand it, the image sequence used in support of this model (Fig 7D and the supplementary video) don't show this at all - rather they seem to show two structures with rather similar potential that undergo a flash in which one suddenly increases a bit whilst the other almost disappears, to gradually reappear later. While it is true that they equilibrate at the end, why should the potential of one decrease so much? The hyperpolarising structure doesn't start off depolarised and then increase potential to equilibrate with its neighbour, but starts off the same and then increases a bit. I find it very hard to understand how this can happen according to the model.

*We thank the reviewer for the positive comments. We have changed the color display in Fig. 7D and its associated movie (showing now images color-coded in "gold" and an additional F/F0 movie) to better show the increase in potential in the hyperpolarizing structure. It is true that the extent of TMRM loss in the depolarizing structure is greater than the extent of TMRM gain in the hyperpolarizing structure, both during the event and after equilibration, but this might reflect the non-linearity of TMRM fluorescence changes as a function of voltage. We are currently attempting to calibrate the TMRM/GFP recordings with valinomycin/K+ to clarify this point. *

**

I have to confess also that I still struggle to understand exactly what was done during the paGFP experiments - it isn't clear to me what areas of the image were flashed and the GFP activated. I dare say I am being dim witted, but I think it needs to be clearer given the time most people are willing to give to reading a paper these days.

*We have added a crosshair on the high-magnification images to identify the region that was irradiated to photoactivate the GFP. *

One of the co-referees doesn't like the pore model, and it has to be said that you have no direct evidence that there is a fusion pore. It would seem reasonable to make it clear that this is speculative. However I would imagine that theoretically you would need some kind of pore opening (according to this model) in order to generate a rapid transient that then relaxes - gradual membrane fusion would presumably just cause gradual equilibration.

*We now stress the speculative nature of the fusion pore and have added a question mark in the final model to highlight this. *

Referee #2:

While the authors have addressed most of the minor concerns, some major issues have not resolved:

*There was not distinction between major and minor concerns in the previous comments from this reviewer. We addressed all the points of the initial review, either by providing explanations and

*clarifications (points 1, 2, 5, 6, and 7) or new data (points 3, 4, and 5). **

- The major concern still present after this round of revision is that there is no direct evidence that the pH flashes are caused by an hypothetical transient pore that resembles gap junctions during a brief mitochondrial fusion event, as proposed by the authors.

**This concern is valid but was not phrased explicitly in the initial report. We agree that we lack direct evidence that a fusion pore forms during mitochondrial fusion. However, the new experiments suggested by the reviewer will not provide such evidence either, as proof of gap junction-like communication will require electrophysiological recordings of apposed mitochondria, very demanding experiments that we believe are clearly outside the scope of this paper. In this study, we provide two independent lines of evidence that diffusion-limiting pores connect mitochondria by showing 1) that pairs of mitochondria are functionally coupled (Fig. 7) and 2) that pH flashes spread without matrix GFP exchange along apposed mitochondria (Fig. 5 and 6). We also provide evidences linking pore opening to fusion by showing that the pH flashes 1) coincide spatially and temporally with fusion events (Fig. 5C) and 2) require OPA1-mediated fusion (Fig. 6). Although indirect, these evidences clearly suggest that OPA1 controls the opening of a fusion pore that functionally couples juxtaposed mitochondria. **

Furthermore, no experiments were performed to show and/or directly quantify inner as well as outer membrane fusion and its correlation to a pH flash, which are necessary to prove the author's hypothesis.

**We would like to refute this comment. Quantifying the extent of inner or outer membrane fusion will neither prove nor invalidate our hypothesis. A fusion pore that resembles a gap junction could form without exchange of IMM proteins or even of OMM proteins. Gathering evidence that mitochondria exchange membrane proteins during a pH flash will reveal whether flash activity is linked to successful fusion events but will not identify the molecular mechanism causing the pH flashes. This experiment will only strengthen the link between flash activity and mitochondrial fusion, which we already have firmly established with genetic manipulations and functional assays. **

This could had been done by PEG cell fusion assays of two cells expressing differentially labeled outer or inner membrane proteins and see how outer and/or inner membrane protein transfer or transient colocalization correlates to a pH flash (as the resolution of confocal fluorescence microscopy is around 200 nm). Therefore, the molecular mechanism causing the pH flashes has not been successfully identified.

**This experiment is problematic in several respects. First, enforcing cell fusion with PEG introduces confounding factors as it promotes lipid mixing. Second, as discussed above, the experiment will not prove or invalidate our model. Colocalization of differentially labeled mitochondria during a flash will essentially provide the same information as Fig. 5C, which shows that pH flashes correlate temporally and spatially with fusion events, while transfer of membrane proteins exchange during a pH flash will link flashes to OMM or IMM fusion but will not identify the molecular mechanism involved. Third, color separation will be problematic as we need to express the SypHer probe in the mitochondrial matrix to record pH flashes. We therefore believe that the potential gain of information that can be obtained by the PEG cell fusion assay is limited and does not justify the experiment. **

-Despite being feasible, experiments acutely modulating mitochondrial fusion and fission events were not performed.

Examples of experimental models currently available and previously published that acutely modulate fusion and fission rates are listed here: Mdivi treatment- Drp1 inhibitor-, nocodazole treatment to prevent mitochondrial movement and thus mitochondrial fusion. Other medium term experiments would be inducible siRNA for Mfns, Opa1, Drp1 or Cre in Mfn1, Mfn2 and Drp1 LoxP cells. However, these experiments would require some additional set up, as some effects could be caused by an alteration in mitochondrial quality control mechanisms and not just by decreased fusion and or fission events. The experimental models currently used in the paper (KO cells) cannot

be used to prove the author's hypothesis, as lack of pH flashes can be caused by mitochondrial dysfunction induced by the genetic modifications used, by altered mtDNA homeostasis,and due to the long term inhibition of inter-mitochondrial fusion and complementation. Therefore, the precise molecular mechanism behind the pHflashes remains elusive.

**Acute modulation of mitochondrial fusion and fission was previously mentioned by the reviewer, but not specifically requested (point # 7). The reviewer now insists on these experiments on the ground that our KO cells cannot be used to prove our hypothesis because they might have dysfunctional mitochondria (a concern that he/she admits also applies to acute manipulations). We would like to stress the benefits of KO models, which provide complete and selective genetic ablation of specific mitochondrial shaping proteins. Loss of pH flashes upon /Opa1/ ablation, complemented by re-expression of OPA1 but not MFN1, genetically links OPA1 to pH flash activity. Since the main function of OPA1 is to mediate IMM fusion, our KO models causally link flash activity to IMM fusion. We agree that long term inhibition of fusion can have non-specific effects, but this concern applies to all chronic models while abrogation of pH flashes was only observed in /Opa1^-/- /cells. As stressed in our initial rebuttal letter, /Opa1^-/- / cells were previously shown to be bioenergetically competent and to retain a normal potential and pH (Gomes et al., Nature Cell Biol. 2011). Furthermore, our study includes acute manipulations of fusion and fission, since we show that transient expression of hFIS1 and of dominant negative DRP mutants alter pH flash propagation (Fig. 5). We are reluctant to use chemicals such as Mdivi and nocodazole, which have pleiotropic effects on cell trafficking, or siRNA that only provide partial protein depletion and can have off-target effects. We agree that the Cre/LoxP system provides a clean and tractable model and we are now generating /Opa1/ floxed mice that will allow conditional ablation of the IMM fusion protein. However, as stated in our initial rebuttal letter, the generation and validation of this cellular model will require a substantial amount of time, and we believe that these cells will be better exploited in a subsequent study. **

If authors do not perform these experiments, I would recommend publishing this manuscript only if author's remove the model proposing that pH flashes are caused by a pore opening. The data only shows that pH flashes are dependent on the electron transport chain function and that modulation of mitochondrial homeostasis by changing fusion and fission proteins can regulate the frequency of these flashes.

**As discussed above, we prefer not to perform these experiments and therefore agree to tune down our conclusions. We now mention in the discussion that the existence of fusion pores electrically connecting mitochondria remains hypothetical and awaits direct electrophysiological proof. However, we would like to keep the model, stressing that it is hypothetical, because we believe that it provides a new and coherent conceptual framework for the existence of the spontaneous fluctuations in mitochondrial potential and pH that have mystified the scientific community for years. Sound experimental research requires models that can be tested, and we believe that our model will advance this field of research by prompting others to perform new and innovative experiments. **

Referee #3:

I believe that the authors have responded well to the criticisms raised. While not all of the answers fully address the reviewers' concerns, I think the authors have made considered efforts to improve the manuscript with additional experimental data and (importantly) significant changes within the text. Some of the experimental approaches requested by the reviewers, are in my opinion, out of the realms of expectation and would take many months and significant resources to complete - and still would not lead to definitive results. With no disrespect to The EMBO Journal (which is a high quality journal), I also think that should the authors obtain results using additional sophisticated experiments, they would have pitched the manuscript to a higher-tier journal. In essence, I believe that the manuscript should be accepted for publication.

I agree that there are inherent issues with using the Opa1 knockout cell line which is known to cause mitochondrial bioenergetic defects. However, the known properties of Opa1 in mitochondrial inner membrane fusion is a strong indicator that this is the protein most likely involved in spreading pH flashes. Importantly, the authors show that the pH flashes are recovered upon Opa1 re-expression. How atractyloside is involved in stimulating pH flashes is something that I expect will lead to others

to investigate.

I favour that the authors keep the model since it is an important summary of the work. The authors clearly state that this is a "proposed mechanism". It will be up to the scientific community to determine whether this is in fact a "pore". Identification of components of other controversial mitochondrial pores (e.g. related to apoptosis, calcium flow and mitochondrial permeability transitions) has aided scientific debate and research studies and I imagine this relatively controversial topic will be similar. A compromise could be to not show a pore that looks like a protein channel but instead place a question mark in a circle/box.**

*We thank the reviewer for the positive comments. As suggested, we now include a question mark in the final model to highlight the speculative nature of the fusion pore. *

© EMBO
